# Acquired Radioresistance Through Adaptive Evolution with Gamma Radiation as Selection Pressure: Increased Expression and Induction of Anti-Stress Genes

**DOI:** 10.3390/ijms26157275

**Published:** 2025-07-28

**Authors:** Takeshi Saito, Hiroaki Terato

**Affiliations:** 1 Division of Radiation Life Science, Institute for Integrated Radiation and Nuclear Science, Kyoto University, 2-1010, Asashiro-nishi, Kumatori-cho, Sennan-gun 590-0494, Osaka, Japan; 2 Department of Radiation Research, Advanced Science Research Center, Okayama University, 2-5-1, Shikata-cho, Kita-ku, Okayama-shi 700-8558, Okayama, Japan; hiroakiterato@okayama-u.ac.jp

**Keywords:** radioresistant bacteria, *Escherichia coli*, adaptive evolution, gene expression changes, anti-stress genes, DNA repair, cell recovery

## Abstract

Elucidating the mechanisms of radioresistance in highly radiotolerant organisms can provide valuable insights into the adaptation and evolution of organisms. However, research has been limited on many naturally occurring radioresistant organisms due to a lack of information regarding their genetic and biochemical characteristics and the difficulty of handling them experimentally. To address this, we conducted an experiment on adaptive evolution using gamma radiation as the selection pressure to generate evolved *Escherichia coli* with gamma radiation resistance approximately one order of magnitude greater than that of wild-type *E. coli*. Gene expressions in all wild-type and evolved radioresistant *E. coli* in the presence or absence of gamma irradiation were analyzed and compared using RNA sequencing. Under steady-state conditions, the genes involved in survival, cell recovery, DNA repair, and response following stress exposure were upregulated in evolved *E. coli* compared with those in wild-type *E. coli*. Furthermore, the evolved *E. coli* induced these genes more efficiently following gamma irradiation and greater DNA repair activity than that in the wild-type *E. coli*. Our results indicate that an increased steady-state expression of various anti-stress genes, including DNA repair-related genes, and their highly efficient induction under irradiation are responsible for the remarkable radioresistance of evolved *E. coli*.

## 1. Introduction

Organisms have evolved to adapt to diverse environments. Certain species survive in environments characterized by extreme temperatures, salt concentrations, acidity, and alkalinity [1,2,3,4,5,6]. Elucidating the adaptation mechanisms of such species to harsh conditions is expected to provide valuable insights into biological evolution and organismal diversity. Among organisms that can tolerate adverse conditions, certain bacteria are highly resistant to ionizing radiation [7]. Understanding the mechanisms underlying the remarkable radioresistance of these bacteria is of great interest in the context of organismal adaptation.

Studies on radioresistant bacteria have mainly focused on *Deinococcus radiodurans*, the first reported radioresistant bacterial species [8]. Various indicators have been used to compare the radioresistance of organisms, with the 37% survival dose—defined as the radiation dose that reduces an organism’s survival rate to 37%—being one of the most commonly used [7]. The 37% survival gamma radiation dose in *D. radiodurans* can reach 7 kGy, over 1000 times greater than that in humans [9]. The mechanisms suggested to drive radioresistance in *D. radiodurans* include high DNA repair activity through mechanisms common to bacteria and specific to *D. radiodurans*, a high antioxidant capacity conferred by low-molecular-weight substances, such as carotenoids and enzymes such as superoxide dismutase and catalase, high concentrations of protein-protective Mn ions in the cytosol, and suppressed diffusion of damaged genomic DNA through aggregated nucleoid structures [7,10,11,12]. However, a consensus on the primary driver of resistance remains elusive.

Radioresistant bacteria other than *D. radiodurans*, such as *Rubrobacter radiotolerans* and *Kineococcus radiotolerans,* have also been isolated from natural environments [7] and are extensively and randomly distributed across the phylogenetic tree [13]. Thus, studies focusing solely on the genus *Deinococcus* are unlikely to provide a complete picture of the mechanisms of radioresistance in these bacteria. However, conducting experimental research on these organisms is particularly challenging due to the limited knowledge of the genetic and biochemical characteristics of various naturally occurring radioresistant bacteria and the difficulties in handling such species.

Laboratory adaptive evolution studies using *Escherichia coli*, a model organism with well-documented genetic and biochemical characteristics, represent a valuable research strategy for elucidating phenotypes such as radioresistance [14,15,16]. However, these studies have mainly focused on phenotypic changes and mutations in structural genes, with little progress made on changes in gene expression [17,18]. This has also been observed in evolutionary studies of *E. coli* subjected to ionizing radiation [19,20,21,22,23,24,25]. Therefore, in the present study, we generated radioresistant *E. coli* through an adaptive evolution experiment with gamma radiation as the selection pressure and analyzed the expression of all genes to clarify the basis of radioresistance and associated evolutionary mechanisms. Our findings showed differentially regulated stress resistance genes in radioresistant *E. coli*, thus implicating these genes as drivers of radioresistance.

## 2. Results

### 2.1. Generation of Radioresistant E. coli via Adaptive Evolution

**Figure 1:**
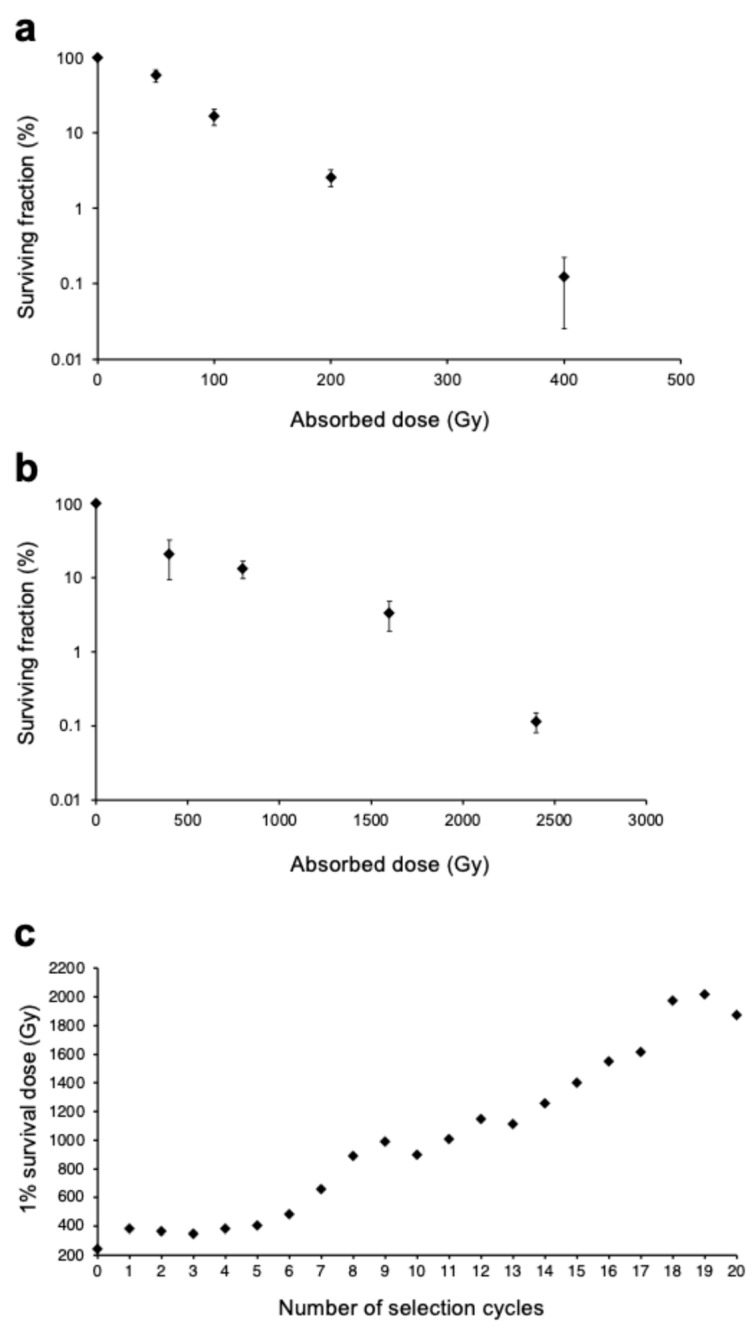
Increased *Escherichia coli* radioresistance through adaptive evolution experiment with gamma radiation as selection pressure. Survival curve of (**a**) gamma-irradiated wild-type *E. coli* and (**b**) gamma-irradiated *E. coli* populations after 20 selection cycles in adaptive evolution experiment. Horizontal axis shows absorbed dose of gamma radiation, and vertical axis shows surviving fraction. Surviving fractions are shown as mean ± standard deviation (SD) from five independent experiments; (**c**) Changes in radioresistance of *E. coli* as adaptive evolution experiment progressed. Horizontal axis shows number of selection cycles, and vertical axis shows 1% survival dose.

To generate radioresistant *E. coli,* cells were cultured in an Luria–Bertani (LB) medium at 37 °C until the early log phase. The culture was resuspended in phosphate-buffered saline (PBS) and irradiated with a 1% survival dose of gamma radiation. Subsequently, the irradiated *E. coli* were cultured in an LB medium, and their sensitivity to gamma radiation was evaluated via a colony formation assay. The selection procedure was repeated 20 times. Figure 1a shows the relationship between the absorbed dose and the surviving fraction of gamma-irradiated wild-type *E. coli*. Based on this survival curve, the 1% survival dose for gamma radiation for wild-type *E. coli* was 240 Gy. The 1% survival dose for the *E. coli* population obtained after 20 selections was 1900 Gy (Figure 1b). Thus, the adaptive evolution experiment yielded an evolved *E. coli* population 7.9 times more resistant to gamma radiation than the wild-type. Figure 1c shows the relationship between the number of selection cycles and the 1% survival dose. For up to five cycles of selection, no substantial change was observed in the resistance of *E. coli* to gamma radiation. However, after the sixth cycle, the *E. coli* radioresistance gradually increased. If only a limited number of genetic changes were involved in the evolution, radioresistance would be expected to increase in a stepwise manner with increasing selection cycles [21]. The gradual increase in radioresistance in the present experiment indicates that multiple genetic changes, including gene expression, were involved in this adaptive evolution.

### 2.2. Alterations in Steady-State Gene Expression Through Evolution

**Figure 2:**
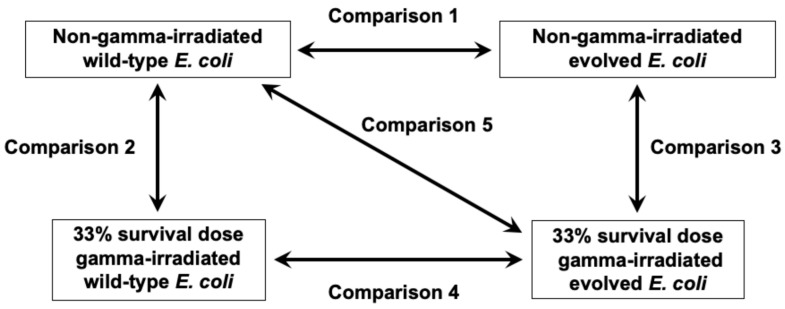
Comparison of cell populations and treatment conditions.

As noted above, multiple genetic changes were involved in the radioresistance acquired through adaptive evolution. Therefore, it is crucial to analyze and compare the expression of all genes in the wild-type and evolved radioresistant *E. coli* populations under various conditions to elucidate the mechanisms involved in the radioresistant phenotype. We used RNA sequencing (RNA-seq) to analyze the steady-state gene expression in the wild-type and evolved radioresistant *E. coli*. We also analyzed the gene expression in these cells after irradiation with a 33% survival dose of gamma radiation (65 Gy for wild-type and 420 Gy for evolved *E. coli*) to clarify their responses to irradiation. The cell populations compared in this study and the treatment conditions are summarized in Figure 2.

**Figure 3:**
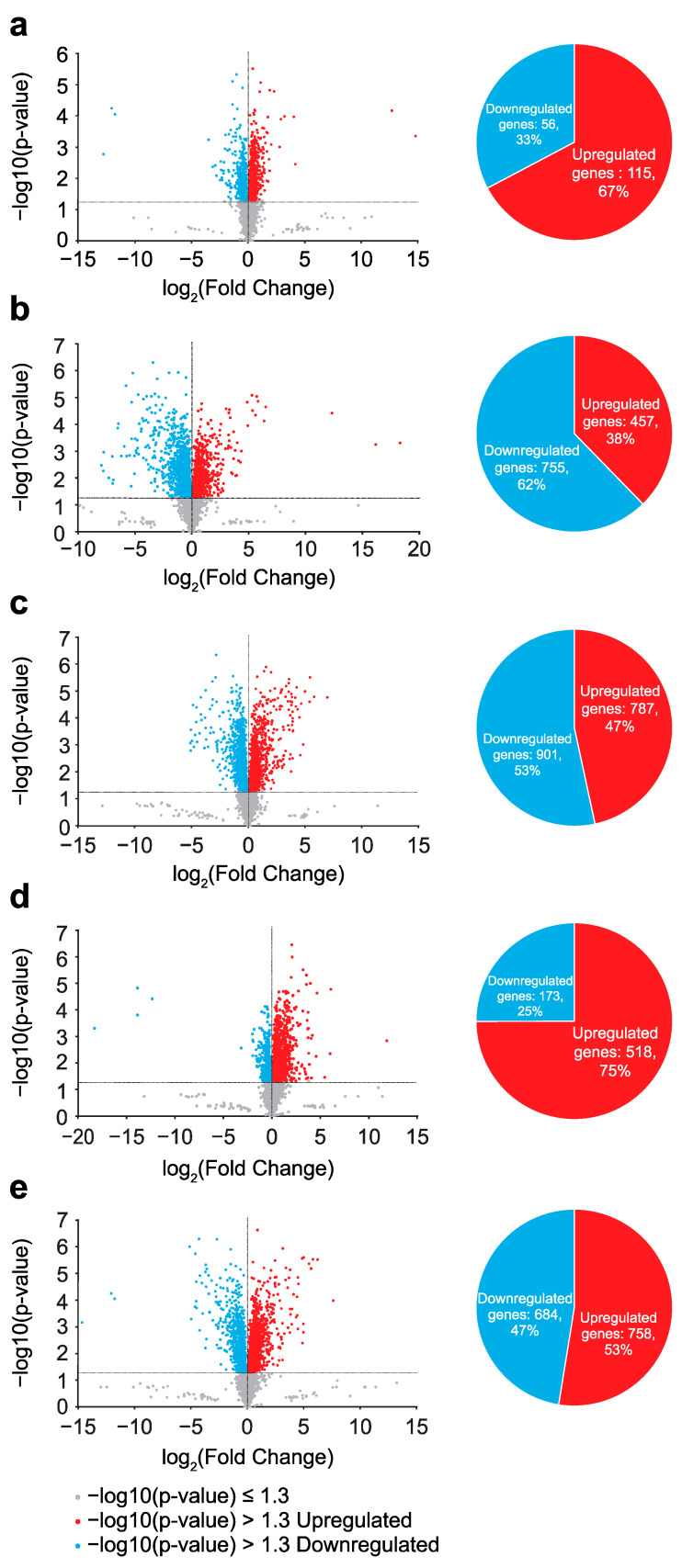
Comparison of gene expression between cell populations. Gene expression differences between (**a**) non-gamma-irradiated evolved and non-gamma-irradiated wild-type *E. coli*; (**b**) gamma-irradiated wild-type and non-gamma-irradiated wild-type *E. coli*; (**c**) gamma-irradiated evolved and non-gamma-irradiated evolved *E. coli*; (**d**) gamma-irradiated evolved and gamma-irradiated wild-type *E. coli*; (**e**) gamma-irradiated evolved and non-gamma-irradiated wild-type *E. coli*. Left panels: volcano plots for each comparison. Horizontal axis shows changes in gene expression (fold change value), and vertical axis shows significance of changes in individual genes (*p*-value). Genes with −log10(*p*-value) > 1.3 are given in color. Red, genes with increased expression; blue, genes with decreased expression. Right panels: number of genes with significantly increased or decreased expression in each comparison with corresponding proportions. Significantly differentially expressed genes (DEGs) were identified using Welch’s *t*-test and multiple testing corrections using Benjamini–Hochberg method (q < 0.05). Red, upregulated genes; blue, downregulated genes.

Initially, gene expression was compared between non-irradiated wild-type and evolved *E. coli* (Figure 2, Comparison 1). Figure 3a (left panel) shows the considerable gene expression differences between the two groups using a volcano plot. Significantly differentially expressed genes (DEGs) were identified using Welch’s *t*-test and multiple testing corrections using the Benjamini–Hochberg method. A total of 115 upregulated and 56 downregulated genes were noted in the evolved compared with wild-type *E. coli* (Figure 3a, right panel).

**Figure 4:**
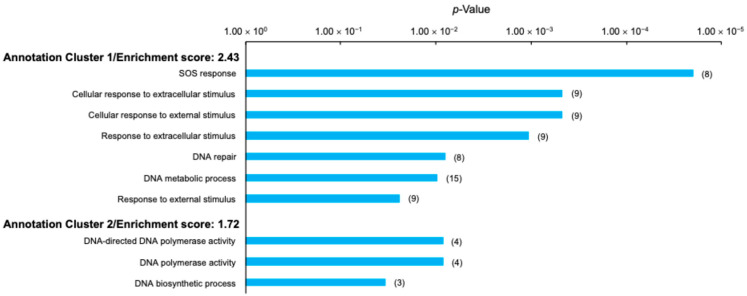
Gene ontology (GO) analysis of genes significantly upregulated in non-gamma-irradiated evolved *E. coli* compared with those in non-gamma-irradiated wild-type *E. coli*. Vertical axis shows Annotation Cluster number, enrichment score for each Annotation Cluster, and GO terms present in each Annotation Cluster. Horizontal axis shows *p*-value for each GO term. Gene counts are shown in parentheses to right of bars.

The results of the gene ontology (GO) analysis of these significant DEGs are summarized in Figure 4 and Supplementary Table S1. Significantly enriched terms, including SOS response, DNA repair, response to stimuli, and DNA metabolism, were present in Annotation Cluster 1 (2.43 enrichment score) and Annotation Cluster 2 (1.72 enrichment score). Thus, at steady state, the genes involved in survival, cell recovery, DNA repair, and response following stress exposure were upregulated in the evolved, compared with wild-type, *E. coli*. The results of the GO analysis for the significantly downregulated genes are summarized in Supplementary Figure S1. At steady state, the genes involved in metabolism and redox were downregulated via evolution compared with wild-type *E. coli*.

Significant DEGs were also subjected to Kyoto Encyclopedia of Genes and Genomes (KEGG) pathway analysis. At steady state, the genes involved in metabolism-related pathways were upregulated in the evolved compared with wild-type *E. coli* (Supplementary Figure S2). Downregulated genes were not significantly enriched in any KEGG pathway terms. These results highlight the differences in steady-state gene expression between wild-type and evolved *E. coli*. Furthermore, the genes involved in survival, cell recovery, DNA repair, and response following stress exposure (hereafter referred to as “anti-stress genes”) were significantly upregulated in evolved *E. coli*.

### 2.3. Gamma Irradiation-Induced Gene Expression Changes in Wild-Type E. coli

Gene expression was compared between non-gamma-irradiated and gamma-irradiated wild-type *E. coli* (Figure 2, Comparison 2). Figure 3b shows gene expression changes in wild-type *E. coli* after gamma irradiation at a 33% survival dose. In total, 457 genes were significantly upregulated, and 755 genes were significantly downregulated by gamma irradiation. The results of the GO analysis for the significantly upregulated genes are summarized in Supplementary Figure S3. No significant enrichment of anti-stress genes was observed among those upregulated by gamma irradiation. In contrast, gamma irradiation upregulated the genes involved in metabolism, ribosome, redox, GTP, and translation. The GO analysis results for the significantly downregulated genes are summarized in Supplementary Figure S4. The genes involved in metabolism, phosphorylation, transport, redox, isomerization, and respiration were downregulated in wild-type *E. coli* following gamma irradiation.

The KEGG pathway analysis indicated that the genes involved in pathways related to metabolism, ribosome, and RNA polymerase were upregulated in gamma-irradiated wild-type *E. coli* (Supplementary Figure S5). In contrast, the downregulated genes were implicated in metabolism, phosphorylation, the two-component system, and chemotaxis (Supplementary Figure S6). These results show that gamma irradiation altered gene expression in wild-type *E. coli*. However, no significant differential regulation of anti-stress genes was observed.

### 2.4. Gamma Irradiation-Induced Gene Expression Changes in Evolved E. coli

Gene expressions were compared between irradiated and non-irradiated evolved *E. coli* (Figure 2, Comparison 3). Figure 3c shows the gene expression changes in the evolved *E. coli* after gamma irradiation at a 33% survival dose. A total of 787 and 901 genes were significantly upregulated and downregulated, respectively. The results of the GO analysis for the significantly upregulated genes are summarized in Supplementary Figure S7. SOS response, DNA repair, and response to stimuli were the GO terms in Annotation Cluster 3 (4.16 enrichment score). Thus, the anti-stress genes were enriched among the upregulated DEGs in the evolved *E. coli* subjected to gamma irradiation. Other enriched terms included metabolism, respiration, redox, metal clustering, ion binding, cytochrome, ribosome, antioxidant, RNA binding, and transport. The GO results for the significantly downregulated genes are summarized in Supplementary Figure S8. Downregulated DEGs were enriched in transport, RNA, ribosome, redox, respiration, metabolism, and membrane structure.

The KEGG pathway analysis indicated that the genes related to metabolism, ribosome, and transport pathways were enriched among those upregulated in the gamma-irradiated evolved *E. coli* (Supplementary Figure S9). In addition, downregulated DEGs were implicated in mismatch repair, DNA replication, metabolism, and homologous recombination (Supplementary Figure S10). These results show that gamma irradiation significantly induced anti-stress genes in the evolved *E. coli*.

### 2.5. Changes in Effect of Gamma Irradiation on Gene Expression Alteration Through Evolution

**Figure 5:**
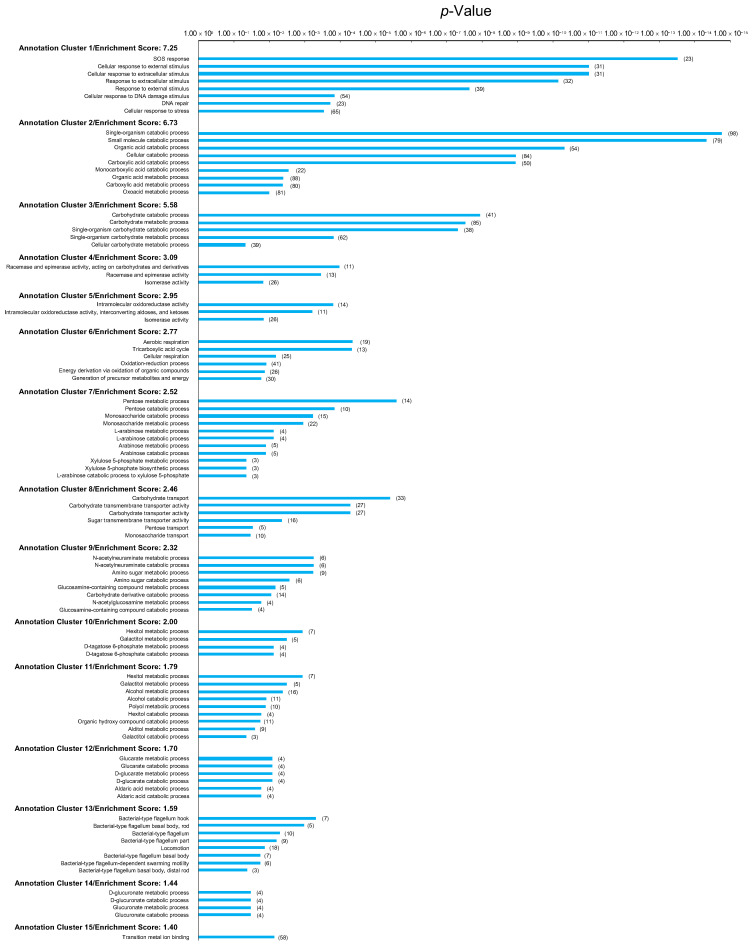
GO analysis of genes significantly upregulated in gamma-irradiated evolved *E. coli* compared with those in gamma-irradiated wild-type *E. coli*. Vertical axis shows Annotation Cluster number, enrichment score for each Annotation Cluster, and GO terms present in each Annotation Cluster. Horizontal axis shows *p*-value for each GO term. Gene counts are shown in parentheses to right of bars.

Gene expressions were compared between the gamma-irradiated wild-type and evolved *E. coli* (Figure 2, Comparison 4). Figure 3d shows the differences in gene expression. Compared with the gamma-irradiated wild-type *E. coli*, 518 genes were significantly upregulated, and 173 genes were significantly downregulated in gamma-irradiated evolved *E. coli*. The results of the GO analysis for the significantly upregulated genes are summarized in Figure 5 and Supplementary Table S2. Upregulated DEGs were significantly enriched for the SOS response, DNA repair, and response to stimuli GO terms in Annotation Cluster 1 (7.25 enrichment score). Thus, anti-stress genes were enriched among those upregulated in the evolved *E. coli* compared with wild-type *E. coli* exposed to gamma irradiation. The upregulated DEGs were also enriched in metabolism, isomerization, redox, respiration, transport, motility, and ion binding. The results of the GO analysis for the significantly downregulated genes in gamma-irradiated evolved versus wild-type *E. coli* are summarized in Supplementary Figure S11. These genes were enriched for RNA and transport.

KEGG pathway analysis revealed the enrichment of upregulated DEGs in the pathways related to metabolism and flagellar structure (Supplementary Figure S12). The analysis for downregulated genes did not detect any significant pathways. These results highlight distinct gamma irradiation-induced gene expression changes between the wild-type and evolved *E. coli*. Furthermore, the expression levels of anti-stress genes in evolved *E. coli* under gamma irradiation were higher than those in wild-type *E. coli*, indicating that the induction efficiency of the anti-stress genes was higher in evolved *E. coli* than in wild-type *E. coli*.

### 2.6. Effects of Evolution and Gamma Irradiation on Gene Expression Changes

Gene expression was also compared between the non-gamma-irradiated wild-type and gamma-irradiated evolved *E. coli* (Figure 2, Comparison 5). Figure 3e shows the gene expression differences between the gamma-irradiated evolved versus non-gamma-irradiated wild-type *E. coli*. A total of 758 and 684 genes were significantly upregulated and downregulated, respectively, in gamma-irradiated evolved *E. coli*.

The results of the GO analysis for the significantly upregulated DEGs are summarized in Supplementary Figure S13. The GO terms for SOS response, DNA repair, and response to stimuli were present in Annotation Cluster 1 (4.70 enrichment score). Thus, anti-stress genes were enriched among the upregulated DEGs in the gamma-irradiated evolved compared with non-gamma-irradiated wild-type *E. coli*. The upregulated genes were also involved in metabolism, ribosome, redox, respiration, cytochrome, metal cluster, and quorum sensing. The results of the GO analysis for the significantly downregulated genes are summarized in Supplementary Figure S14. The genes involved in metabolism, ion binding, redox, respiration, transport, membrane structure, and RNA were enriched in the genes whose expression was decreased in gamma-irradiated evolved compared with non-gamma-irradiated wild-type *E. coli*.

The KEGG pathway analysis indicated that upregulated DEGs were involved in pathways related to metabolism, transport, and ribosome (Supplementary Figure S15), whereas the downregulated DEGs were implicated in metabolism and the two-component system (Supplementary Figure S16). Taken together, these results confirm that the expression of anti-stress genes was significantly increased in gamma-irradiated evolved compared with non-gamma-irradiated wild-type *E. coli*.

### 2.7. Changes in Expression Levels of Anti-Stress Genes Induced by Evolution and Gamma Irradiation

As described above, the GO and KEGG pathway analyses revealed qualitative changes in the expression of anti-stress genes. To analyze the changes in the expression levels of these anti-stress genes quantitatively, we performed hierarchical clustering and quantitative real-time reverse transcription polymerase chain reaction (qRT-PCR) analyses.

**Figure 6:**
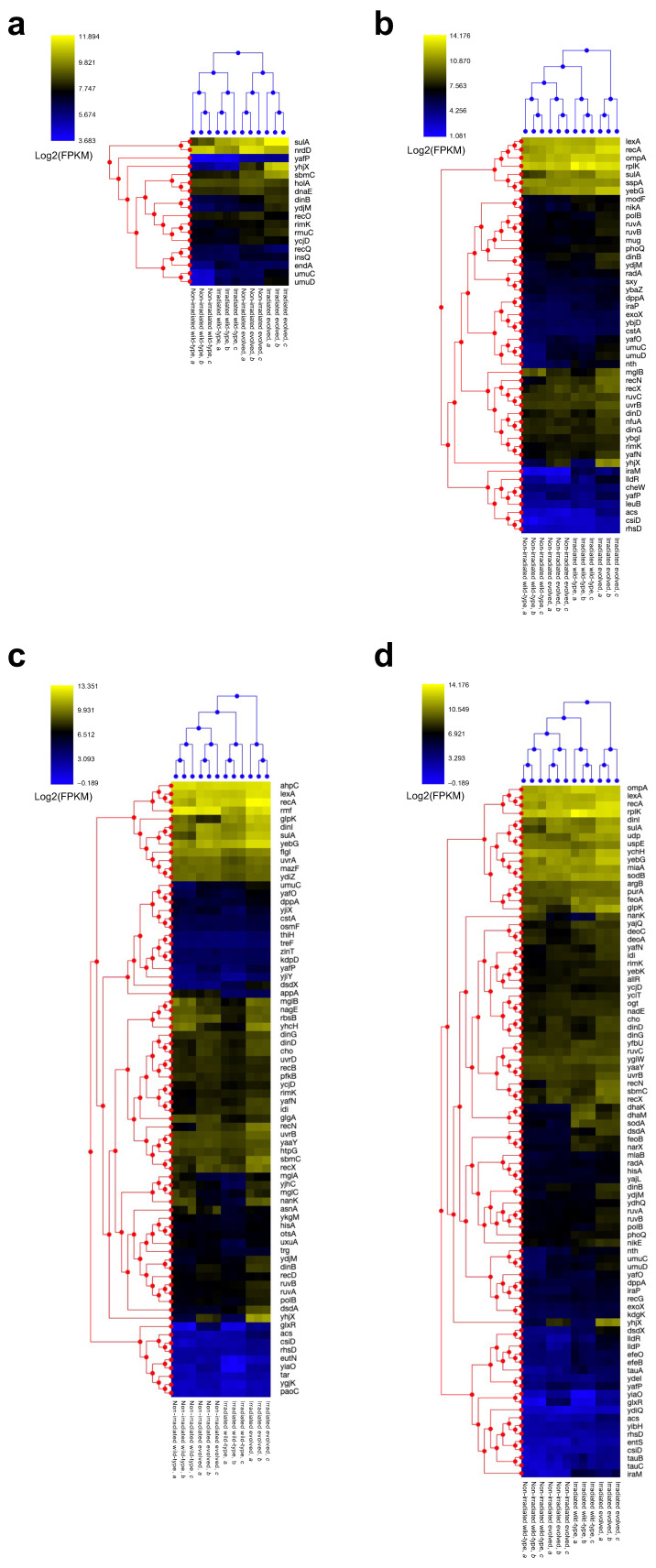
Heat map of hierarchical clustering analysis. Color pattern changes from blue to yellow with increasing gene expression; hierarchical clustering analysis of expression levels of genes (**a**) enriched in GO analysis for upregulated DEGs in non-gamma-irradiated evolved compared with non-gamma-irradiated wild-type *E. coli*; (**b**) belonging to Annotation Cluster 3 from GO analysis for upregulated DEGs in gamma-irradiated evolved compared with non-gamma-irradiated evolved *E. coli*; (**c**) belonging to Annotation Cluster 1 from GO analysis for upregulated DEGs in gamma-irradiated evolved compared with gamma-irradiated wild-type *E. coli*; (**d**) belonging to Annotation Cluster 1 from GO analysis for upregulated DEGs in gamma-irradiated evolved compared with non-gamma-irradiated wild-type *E. coli*. Horizontal axis shows samples. The letters a, b, and c shown after the sample names denote distinct samples derived from three biological replicates. Vertical axis shows genes. The node points on the red and blue dendrograms are indicated by dots.

First, a hierarchical clustering analysis was performed on the expression levels of the genes enriched in the GO analysis of the DEGs upregulated in non-gamma-irradiated evolved versus non-gamma-irradiated wild-type *E. coli* (Figure 2; Comparison 1, Figure 6a). Three samples within the target group formed clusters. The distance between the non-gamma-irradiated and the gamma-irradiated wild-type *E. coli* sample clusters was small, as was that between the non-gamma-irradiated and gamma-irradiated evolved *E. coli* clusters. 

Next, a hierarchical clustering analysis was performed on the expression levels of the genes belonging to Annotation Cluster 3 in the GO analysis of the DEGs upregulated in gamma-irradiated versus non-gamma-irradiated evolved *E. coli* (Figure 2; Comparison 3, Figure 6b). Three samples within the target group formed clusters. The non-gamma-irradiated wild-type *E. coli* sample cluster was closest to that of the non-gamma-irradiated evolved *E. coli* samples. The second-smallest distance was found for the gamma-irradiated wild-type *E. coli* sample cluster, whereas that from the gamma-irradiated evolved *E. coli* samples was the largest.

Next, we performed a hierarchical clustering analysis on the expression levels of the genes belonging to Annotation Cluster 1 in the GO analysis of the upregulated DEGs in gamma-irradiated evolved versus wild-type *E. coli* (Figure 2; Comparison 4, Figure 6c). Three samples within the target group formed clusters. The cluster of non-gamma-irradiated wild-type *E. coli* samples had the smallest distance from that of the non-gamma-irradiated evolved *E. coli* samples, followed by the cluster of gamma-irradiated wild-type *E. coli* samples, whereas the largest distance was from the cluster of gamma-irradiated evolved *E. coli*.

Finally, we performed a hierarchical clustering analysis on the expression levels of the genes belonging to Annotation Cluster 1 in the GO analysis for the DEGs whose expression was increased in gamma-irradiated evolved versus non-gamma-irradiated wild-type *E. coli* (Figure 2; Comparison 5, Figure 6d). Again, the three samples formed clusters. The non-gamma-irradiated wild-type *E. coli* sample cluster was the closest to that of the non-gamma-irradiated evolved *E. coli* samples, followed by the gamma-irradiated wild-type *E. coli* sample cluster, whereas the largest distance was noted for the gamma-irradiated evolved *E. coli* samples. These results indicated the following ranking regarding anti-stress gene expression: non-gamma-irradiated wild-type *E. coli* < gamma-irradiated wild-type *E. coli* ≈ non-gamma-irradiated evolved *E. coli* < gamma-irradiated evolved *E. coli*.

**Figure 7:**
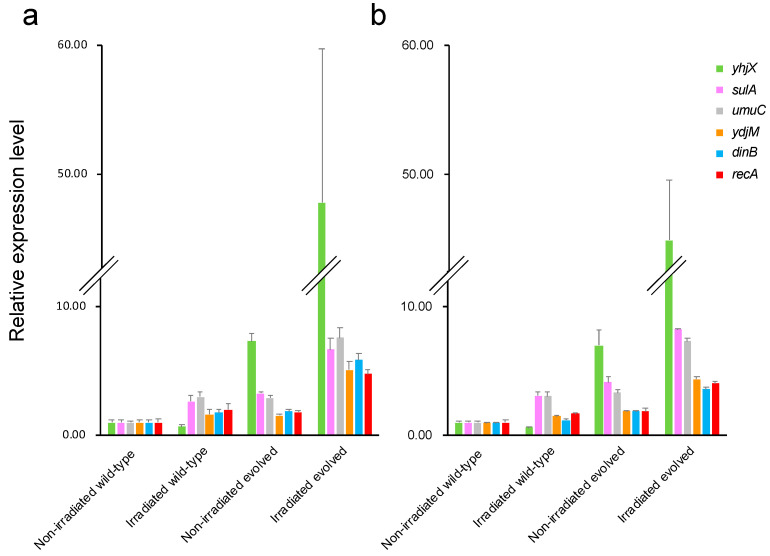
Quantification of expression levels of anti-stress genes. (**a**) Quantification via quantitative real-time reverse transcription polymerase chain reaction (qRT-PCR); (**b**) quantification via RNA sequencing (RNA-seq). Horizontal axis shows samples, and vertical axis shows relative expression levels of each gene. Green, pink, gray, orange, blue, and red bars indicate expression levels of *yhjX*, *sulA*, *umuC*, *ydjM*, *dinB*, and *recA*, respectively.

We used qRT-PCR to quantify the expression levels in each sample of the five genes that were analyzed among all comparison groups in the hierarchical clustering analysis, which differed the most between the non-gamma-irradiated wild-type and the gamma-irradiated evolved *E. coli* (*yhjX*, *sulA*, *umuC*, *ydjM*, and *dinB*), as well as those of *recA*, which has a critical function in DNA double-strand break repair. Figure 7 shows the expression levels determined via qRT-PCR and RNA-seq, which were in good agreement, validating the results obtained via the latter method. The expression levels of these genes ranked as follows among the groups: non-gamma-irradiated wild-type *E. coli* < gamma-irradiated wild-type *E. coli* ≈ non-gamma-irradiated evolved *E. coli* < gamma-irradiated evolved *E. coli*. Anti-stress genes exhibited pronounced upregulation in evolved versus wild-type *E. coli*, with a further increase after induction under gamma irradiation.

### 2.8. Comparison of Protective and Repair Activities Against Gamma Irradiation-Induced Genomic DNA Damage Between Wild-Type and Evolved E. coli

Genomic DNA damage is the main cause of radiation-induced cell death [26]. To assess whether an enhanced protective capacity of genomic DNA is involved in the high radioresistance of evolved *E. coli*, we compared genomic DNA damage in wild-type and evolved *E. coli* irradiated with 0–400 Gy of gamma radiation using the static field gel electrophoresis (SFGE) method. The degree of DNA double-strand breaks (DNA damage) was evaluated based on the proportion of DNA released from the well to the total amount of DNA electrophoresed (hereafter referred to as the “proportion”). No significant difference in the degree of DNA double-strand breaks induced by gamma irradiation was observed between the wild-type and evolved *E. coli* (Supplementary Figure S17), indicating a lack of evidence of enhanced DNA protection in evolved *E. coli*.

**Figure 8:**
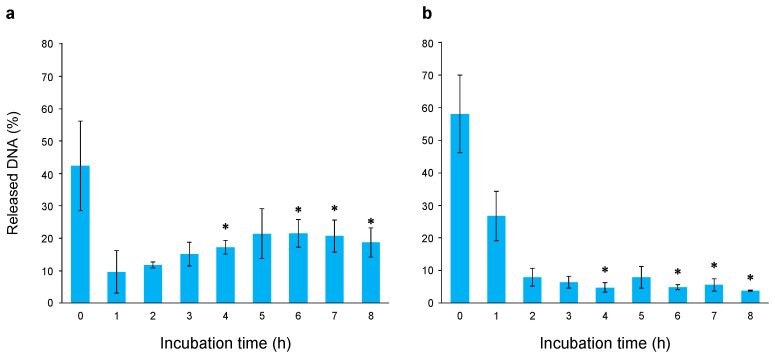
Repair of genomic DNA after gamma irradiation. (**a**) Changes in degree of DNA damage relative to incubation time in wild-type *E. coli*; (**b**) changes in degree of DNA damage relative to incubation time in evolved *E. coli*. Cells irradiated with 2.5 kGy of gamma radiation were shake-incubated in Luria–Bertani (LB) medium at 37 °C. Incubated cells were aliquoted every hour for 0–8 h, and the degree of double-strand breaks in genomic DNA was evaluated using the SFGE method. Horizontal axis shows incubation time, and vertical axis shows proportion of DNA released from well to total amount of DNA. * Samples with significantly different proportions of released DNA relative to total amount of DNA between wild-type and evolved *E. coli* (*p* < 0.05).

We then examined the possibility that the enhanced repair capacity of genomic DNA conferred high radioresistance in the evolved *E. coli*. Wild-type and evolved *E. coli* were irradiated with 2.5 kGy of gamma radiation, followed by shaking incubation of each cell in an LB medium at 37 °C. The incubated cells were aliquoted every hour for 0–8 h, and the degree of double-strand breaks in genomic DNA was evaluated using the SFGE method. No logarithmic growth occurred in any of the cells after up to 8 h of incubation (Supplementary Figure S18), showing that changes in the degree of DNA double-strand breaks were not attributable to cell proliferation. Figure 8 shows the differences between the wild-type and evolved *E. coli* at different incubation times after gamma irradiation. At 4–8 h of incubation (except for 5 h), the proportion of evolved *E. coli* was significantly lower than that of wild-type *E. coli* (*p* < 0.05). For example, the proportion of evolved *E. coli* at 8 h of incubation was 3.9%, whereas that of wild-type *E. coli* was 18.7%. In evolved *E. coli*, the proportion after 1 h of incubation was significantly decreased compared with that at 0 h (*p* < 0.05). In wild-type *E. coli*, there was no significant difference between the proportions at 0 h and after 1 h of incubation. These results show that the DNA double-strand break repair activity of evolved *E. coli* was significantly upregulated compared with that of wild-type *E. coli*, indicating enhanced DNA repair as the basis of high radioresistance in evolved *E. coli*.

## 3. Discussion

In the present study, we performed an adaptive evolution experiment with gamma radiation as the selection pressure and generated an evolved radioresistant *E. coli* population that was 7.9 times more resistant to gamma radiation than wild-type *E. coli*. Our subsequent analyses revealed the following: (1) Steady-state gene expression differed between wild-type and evolved *E. coli*. The genes involved in survival, cell recovery, DNA repair, and response following stress exposure (anti-stress genes) were significantly upregulated in evolved *E. coli* compared with wild-type *E. coli*. (2) The effect of gamma irradiation on gene expression differed between wild-type and evolved *E. coli*. The induction efficiency of anti-stress genes by gamma irradiation in the evolved *E. coli* was greater than that in wild-type *E. coli*. (3) The steady-state expression levels of anti-stress genes were increased in the evolved compared with the wild-type *E. coli*, with further induction by gamma irradiation. (4) Enhanced DNA repair activity was primarily involved in the high radioresistance of the evolved *E. coli*. These results indicate that the increased steady-state expression of numerous anti-stress genes and their highly efficient induction by gamma irradiation are, at least in part, responsible for the radioresistance of the evolved *E. coli*. Our study offers insights into the molecular basis of radioresistance, biological defense mechanisms, and their evolution in bacteria and other organisms. These findings may contribute to applied research regarding evolution in response to external stress, such as elucidating the mechanism of emergence of radioresistant cells during radiotherapy and the mechanism of evolution of drug-resistant microorganisms.

Several studies have analyzed radioresistant *E. coli* generated via adaptive evolution experiments using electromagnetic ionizing radiation (gamma and X-radiation) as the selection pressure [19,20,21,22,23]. Recently, electron beams have also been employed as a selection pressure, triggering a molecular response mechanism distinct from that in response to electromagnetic ionizing radiation [24,25]. However, studies on the radioresistance of evolved *E. coli* have mainly focused on the phenotypic changes and mutations in structural genes. In the present study, we comprehensively analyzed gene expression changes in *E. coli* that evolved radioresistance under gamma radiation and found striking differences compared with wild-type *E. coli*. Byrne et al. previously analyzed the expression profiles of evolved radioresistant *E. coli* generated through an adaptive evolution experiment with gamma radiation and reported little change in the steady-state expression profile of evolved versus wild-type *E. coli* [22]. However, they only analyzed two clonal populations. In our study, gene expression was analyzed in the evolved *E. coli* population. Evolution can be defined as a phenomenon in which the characteristics of a population change over generations [27]. This definition highlights the importance of analyzing genetic changes in the entire evolved population. Therefore, various studies have explored gene expression in cell populations subjected to adaptive evolution [14,17,18,28]. We believe that this difference in experimental approach underpins the discrepancies between our findings and those of Byrne et al. [22]. Organisms respond to external stresses such as radiation in various ways [29]. Thus, irradiation-induced gene expression could provide valuable insights into the radioresistance mechanism of evolved *E. coli*. To the best of our knowledge, this is the first study to comprehensively analyze post-irradiation gene expression changes in radioresistant *E. coli* generated by adaptive evolution.

Anti-stress genes are induced by damaging stimuli, such as DNA damage, as observed during the SOS response. Survival and cell recovery after irradiation through processes such as DNA repair are promoted by genes that include *recA*, *recB*, *recO*, *recQ*, *ruvA*, and *uvrA*; cell division inhibition by *sulA*; and translesion replication mediated via *dinB*, *umuC*, and *umuD*. Furthermore, genes such as *dinB*, *umuC*, and *umuD* likely serve as mutators, increasing the diversity and fitness of the population. The gradual increase in radioresistance after six cycles of selection (Figure 1c) indicates that genetic changes affecting mutation rates may have occurred at this point, promoting a pronounced subsequent increase in population fitness, i.e., radioresistance.

In addition to gene expression changes directly involved in survival, cell recovery, DNA repair, and response following stress exposure, we also observed considerable changes in the expression of genes involved in metabolism, redox, and transport. In the radioresistant bacterium *D. radiodurans*, the inhibition of protein oxidation by the regulation of the redox state through accumulated intracellular Mn ions and by low-molecular-weight antioxidant metabolites contributes to radioresistance [30,31,32,33]. Furthermore, we previously demonstrated that antioxidants, such as specialized carotenoid molecules, are involved in the radioresistance of several radioresistant bacteria, owing to their antioxidant activity [34,35,36,37,38]. Irradiation-induced protein oxidation in one isolate of evolved radioresistant *E. coli* generated via an adaptive evolution experiment with electron beams was lower than that in wild-type *E. coli* [25]. Moreover, metabolic changes may contribute to increased radioresistance in *E. coli* generated through an adaptive evolution experiment with gamma radiation [23]. In our study, changes in the expression of these genes may have optimized the intracellular environment for survival, cell recovery, DNA repair, and biomolecular protection after irradiation through the regulation of metabolism, ion concentration, redox, and antioxidant activity.

A KEGG pathway enrichment analysis of the genes downregulated in gamma-irradiated compared with non-gamma-irradiated evolved *E. coli* revealed enrichment in the pathways related to mismatch repair (Supplementary Figure S10). This downregulation of mismatch repair-related genes, along with changes in *dinB*, *umuC*, and *umuD* expression, may increase the mutation rate and thus promote population fitness. The KEGG analysis also revealed the enrichment of genes belonging to homologous recombination-related pathways (Supplementary Figure S10). Their expression levels were decreased in gamma-irradiated compared with non-gamma-irradiated evolved *E. coli* (Supplementary Figure S19a). However, a GO analysis indicated increased DNA repair gene expression (Supplementary Figure S7). Gamma irradiation upregulated the expression levels of many genes involved in homologous recombination repair, including *recA*, *recN*, *ruvA*, and *ruvB*, in evolved *E. coli* (Figure 6b). Furthermore, the expression levels of all homologous recombination genes, highlighted by the KEGG pathway analysis, increased in non-gamma-irradiated evolved versus wild-type *E. coli* (Supplementary Figure S19b). These results indicate that gamma irradiation induced the downregulation of several homologous recombination-associated genes, which were already upregulated at a steady state in evolved *E. coli* (compared with wild-type *E. coli*). Thus, the optimization of the balance of expression of homologous recombination-associated genes in evolved *E. coli* may contribute to its high homologous recombination repair activity.

This study also compared gene expression between evolved *E. coli* irradiated with 65 Gy of gamma radiation (33% survival dose in wild-type *E. coli*) and non-gamma-irradiated evolved *E. coli*. This comparison revealed no significant DEGs. Considering that the expression of many genes in wild-type *E. coli* was significantly altered by 65 Gy of gamma irradiation (Figure 3b), our findings highlight striking differences in the effect of gamma irradiation on gene expression between the wild-type and evolved *E. coli*. Evolved *E. coli* is likely able to maintain homeostasis even under 65 Gy of gamma irradiation by relying on anti-stress genes that are already upregulated in the steady state. This may also represent an overall optimization of gene expression for environmental adaptation.

Although these alterations in gene expression are thought to be driven by changes in the genomic sequence, the possibility that they are caused by epigenetic changes cannot be completely discarded. To clarify the mechanism of gene expression regulation at the transcriptional level, all genomic sequences, focusing on the transcription regulatory region, between wild-type and evolved *E. coli*, should be compared, and an epigenetic analysis using long-read sequencing or other methods should be performed. In addition, a proteome analysis should be performed to examine the possibility of regulation at the post-transcriptional level. An analysis of the mutations detected by genome sequencing and the activity of DNA repair enzymes with mutations would also provide valuable information for elucidating radioresistance in evolved *E. coli*. A kinetic analysis of gene expression after gamma irradiation will be useful for elucidating the stress response of evolved *E. coli*. An analysis of the resistance of evolved *E. coli* to stresses other than gamma irradiation, such as various types of radiation and oxidative stress, may provide additional insight into the evolution of stress resistance in organisms. How the phenotype acquired by evolved *E. coli* affects its fitness under natural conditions is important in the process of further evolution, and it is desirable to clarify it by culturing evolved *E. coli* and wild-type *E. coli* under competitive conditions.

Even when evolution proceeds under the same selection pressure, ultimately resulting in equivalent phenotypes, different molecular mechanisms of evolution are expected to act on different populations owing to stochastic effects. In adaptive evolution experiments with an electron beam as the selection pressure, several evolved *E. coli* lineages have been identified via mutation analysis, following unique evolutionary paths [24,25]. In the present study, we analyzed changes in gene expression and DNA repair activity in evolved *E. coli* populations resulting from a single adaptive evolution experiment. Although the analysis of evolutionary mechanisms in a single population can reveal one aspect of the mechanism under specific selection pressures, this approach fails to recapitulate the diversity of potentially involved evolutionary mechanisms. Therefore, further adaptive evolution experiments should be conducted to generalize the evolutionary mechanisms driving radioresistance and the biological defenses driven by changes in gene expression in bacteria and other organisms.

Gene expression changes and their overall optimization are thought to play an important role in phenotypic evolution [18,39,40]. The present study revealed that radioresistant *E. coli* evolved under radiation as the selection pressure exhibited increased steady-state expression and highly efficient induction of anti-stress genes, including cell recovery and DNA repair genes, in addition to the accumulation of mutations. These results indicate that increased expression and induction efficiency of many anti-stress genes can greatly enhance radioresistance and bioprotective activity in bacteria and other organisms. Furthermore, alterations in the expression of various genes likely contribute to the evolution of defense systems against radiation and other stresses during early evolutionary stages. To provide a more precise explanation of the basic radioresistance and biological defense of bacteria and other organisms and the evolutionary mechanisms involved, replicated adaptive evolution experiments with multiple populations should be conducted, and the number of generations subjected to selection should be increased. Moreover, phenotypes, such as radioresistance, and gene expression in evolving populations at each evolutionary stage should be analyzed to clarify the effects of gene expression changes on the phenotype.

## 4. Materials and Methods

### 4.1. Sensitivity of E. coli Cells to Gamma Irradiation

*E. coli* K-12 MG1655, which was stored in our laboratory and has never been exposed to radiation, was used in this study [41]. *E. coli* was cultured in an LB medium (Lennox L Broth, L7275) (Sigma-Aldrich, St. Louis, MO, USA) at 37 °C with shaking (200 rpm) until the early log phase was reached. One milliliter of the culture was centrifuged at 4000× *g* for 10 min at 20 °C. The supernatant was removed, and 1 mL of PBS was added to suspend the *E. coli*. The *E. coli* suspension was irradiated with an appropriate dose of gamma radiation at 22 Gy/min at 20 ± 3 °C. Gamma irradiation was performed at the Co-60 Gamma-ray Irradiation Facility of the Institute for Integrated Radiation and Nuclear Science, Kyoto University. The irradiated *E. coli* suspension was appropriately diluted with PBS, and the diluted solution was plated on LB agar plates. After the plates were incubated at 37 °C for 12 h, the generated colonies were counted, and the surviving fraction was calculated to obtain a survival curve (colony formation assay).

### 4.2. Adaptive Evolution Experiment with Gamma Radiation as Selection Pressure

*E. coli* was cultured in an LB medium at 37 °C with shaking (200 rpm) until the early log phase. The culture was diluted with PBS and plated on LB agar plates. The plates were incubated at 37 °C for 12 h, and one of the resulting colonies was selected for subsequent experiments.

The procedure described hereafter was repeated 20 times. *E. coli* was cultured in LB medium at 37 °C with shaking (200 rpm) until the early log phase. One milliliter of the culture was centrifuged at 4000× *g* for 10 min at 20 °C. The supernatant was removed, and 1 mL of PBS was added to suspend the *E. coli*. The *E. coli* suspension (approximately 2 × 10^8^ cells) was irradiated with a 1% survival dose of gamma radiation obtained from the survival curve at 22 Gy/min at 20 ± 3 °C. The irradiated *E. coli* suspension was added to 100 mL of LB medium, and the cells were cultured at 37 °C with shaking (200 rpm) until the early stationary phase. Glycerol stocks of the resulting *E. coli* population were prepared, and their resistance to gamma radiation was analyzed based on colony formation.

### 4.3. Preparation of Cells for Gene Expression Analysis

*E. coli* was cultured in an LB medium at 37 °C with shaking (200 rpm) until the early log phase. Twenty milliliters of the culture were centrifuged at 2000× *g* for 20 min at 20 °C. The supernatant was removed, and 5 mL of PBS was added to suspend the *E. coli*. The *E. coli* suspension was irradiated with 0, 65, and 420 Gy of gamma radiation at 22 Gy/min at 20 ± 3 °C. The irradiated *E. coli* suspension was added to 200 mL of LB medium and incubated at 37 °C for 2 h with shaking (200 rpm). After incubation, the *E. coli* suspension was immediately cooled on ice and centrifuged at 4000× *g* for 10 min at 4 °C. The supernatant was then removed to obtain cell pellets.

### 4.4. Analysis of Gene Expression via RNA-Seq

Total RNA was extracted from *E. coli* pellets using RNAiso Plus (Takara Bio Inc., Kusatsu, Japan). The extracted RNA was purified using NucleoSpin RNA Clean-up XS (Macherey-Nagel, Düren, Germany). RNA concentrations were measured using a NanoDrop 1000 spectrophotometer (Thermo Fisher Scientific, Waltham, MA, USA). The quality of the prepared RNA was confirmed using an Agilent 2100 Bioanalyzer (Agilent Technologies, Santa Clara, CA, USA). The rRNA was removed from purified RNA using the Ribo-Zero Magnetic Kit (Gram-Negative Bacteria) (Illumina, San Diego, CA, USA). Sequencing libraries were prepared using the TruSeq Stranded mRNA Sample Prep Kit (Illumina). These procedures were performed in an RNase-free environment. The quality of the sequencing library was confirmed using an Agilent 2100 Bioanalyzer. A sequence analysis of the libraries was performed using a NovaSeq 6000 (Illumina), NovaSeq 6000 S4 Reagent Kit (Illumina), and NovaSeq Xp 4-Lane Kit (Illumina). The sequence depth was 3000 ×, and the read length was 2 × 150 bp. Read sequences were mapped to the GCF_000005845.2 *E. coli* K-12 reference genome (ftp://ftp.ncbi.nlm.nih.gov/genomes/all/GCF/000/005/845/GCF accessed on 22 August 2018) using STAR ver. 2.5.3a [42]. Fragments per kilobase of transcript per million mapped fragments (FPKMs) were calculated using Genedata Profiler Genome ver. 11.0.8 (Genedata, Basel, Switzerland). All kits, equipment, and software were used according to the manufacturer’s instructions. To exclude noise, gene data with FPKM values < 1 for all samples among the comparisons were filtered and removed [43]. The obtained FPKMs were used to compare gene expressions. DEGs were identified via Welch’s *t*-test and multiple testing corrections using the Benjamini–Hochberg method [44]. Genes with q < 0.05 were considered significant DEGs.

### 4.5. GO, KEGG Pathway, and Hierarchical Clustering Analyses

DEGs were subjected to GO and KEGG pathway enrichment analyses using Database for Annotation, Visualization and Integrated Discovery (DAVID) ver. 6.8 [45]. Functional annotation clustering in GO analysis was performed in FAT mode for the biological process, cellular component, and molecular function categories. A modified Fisher’s exact test was used to assess significance in the DAVID analyses, and comparisons with *p* < 0.05 or Enrichment Score ≥ 1.3 were considered significantly different. A hierarchical clustering analysis of the gene expressions among samples was performed using WebMeV [46].

### 4.6. qRT-PCR Analysis

Total RNA from the cells was prepared as described for RNA-seq. The resulting total RNA was reverse-transcribed using a SuperScript VILO cDNA Synthesis Kit (Thermo Fisher Scientific). The resulting cDNA was amplified using TaqMan PreAmp Master Mix (Thermo Fisher Scientific). Real-time PCR was performed using amplified cDNA, TaqMan Universal Master Mix II, no UNG (Thermo Fisher Scientific), a Custom TaqMan Gene Expression Assay for each gene (Thermo Fisher Scientific), a BioMark 48.48 Dynamic Array (Standard BioTools Inc., South San Francisco, CA, USA), and a real-time PCR system/BioMark System (Standard BioTools). All kits and equipment were used according to the manufacturer’s instructions. Assay IDs, primer sequences, and probe sequences for each Custom TaqMan Gene Expression Assay are summarized in Supplementary Table S3. The temperature cycle for the real-time PCR was 50 °C for 2 min and 95 °C for 10 min, followed by 40 cycles of 95 °C for 15 s and 60 °C for 1 min. Relative gene expression levels were calculated via the 2^−ΔΔCt^ method (ABI User Bulletin #2) [47] using *gyrA* [Entrez Gene ID: 946614] as the internal reference gene [48]. PCR template concentrations were prepared to enable quantification via the 2^−ΔΔCt^ method such that the amplification efficiencies among the compared genes were equivalent.

### 4.7. Analysis of DNA Protective Activity

*E. coli* was cultured in an LB medium at 37 °C with shaking (200 rpm) until the early log phase. One milliliter of the culture was centrifuged at 4000× *g* for 10 min at 4 °C. The supernatant was removed, and 1 mL of PBS was added to suspend the *E. coli*. The *E. coli* suspension was irradiated with 0, 50, 100, 200, and 400 Gy gamma radiation at 22 Gy/min on ice. The irradiated *E. coli* suspension was centrifuged at 4000× *g* for 10 min at 4 °C, and the supernatant was removed to obtain cell pellets. The degree of double-strand breaks in the genomic DNA (degree of DNA damage) in the resulting cells was evaluated using the SFGE method [49] as follows: *E. coli* cells (5 × 10^7^ CFU) were embedded in 1% agarose gel plugs (SeaPlaque GTG agarose; Cambrex Corporation, East Rutherford, NJ, USA). The plugs were shake-incubated in a lysis solution (1% Sarcosyl, FUJIFILM Wako Pure Chemical Corporation, Osaka, Japan; 0.2% Proteinase K, FUJIFILM Wako Pure Chemical Corporation; 50 mM Tris-HCl [pH 7.5], and 25 mM EDTA) at 50 °C and 150 rpm for 16 h. The plugs were washed four times (2 h per wash) in TE buffer (10 mM Tris-HCl (pH 8.0) and 1 mM EDTA) and inserted into wells of 0.6% agarose gel (pulsed-field certified agarose) (Bio-Rad Laboratories, Hercules, CA, USA). The gels were electrophoresed in TBE buffer (89 mM Tris, 89 mM boric acid, and 2 mM EDTA) at a constant current of 17 mA/gel for 20 h using an electrophoresis apparatus AE-6111 (ATTO Corporation, Tokyo, Japan). The gels were stained with ethidium bromide (2.5 µg/mL; FUJIFILM Wako Pure Chemical Corporation) in a TBE buffer and then washed twice with autoclaved water (5 h per wash). Gel images were captured under a UV transilluminator using an E-graph AE-9000 (ATTO) gel-imaging system. The proportion of the amount of DNA released from the well via fragmentation to the total amount of DNA electrophoresed was analyzed using the image analysis software ImageJ ver. 1.53 (https://imagej.net/ij/index.html accessed on 8 February 2022).

### 4.8. Analysis of Cell Proliferation After Gamma Irradiation

*E. coli* was cultured in an LB medium at 37 °C with shaking (200 rpm) until the early log phase. Twelve milliliters of the culture were centrifuged at 2000× *g* for 20 min at 4 °C. The supernatant was removed, and 12 mL of PBS was added to suspend the *E. coli*. The *E. coli* suspension was irradiated with 2.5 kGy of gamma radiation at 22 Gy/min on ice. The irradiated *E. coli* suspension was centrifuged at 2000× *g* for 20 min at 4 °C. The supernatant was removed, and 12 mL LB medium was added to resuspend the cells. The resuspension was incubated at 37 °C with shaking (200 rpm). From 0 to 10 h, 1 mL of the *E. coli* suspension was aliquoted at the appropriate times, and the turbidity (optical density) was measured at 600 nm.

### 4.9. Analysis of DNA Repair Activity

*E. coli* was cultured in an LB medium at 37 °C with shaking (200 rpm) until the early log phase. Twelve milliliters of the culture were centrifuged at 2000× *g* for 20 min at 4 °C. The supernatant was removed, and 12 mL of PBS was added to suspend the *E. coli*. The *E. coli* suspension was irradiated with 2.5 kGy of gamma radiation at 22 Gy/min on ice. The irradiated *E. coli* suspension was centrifuged at 2000× *g* for 20 min at 4 °C. The supernatant was removed; 12 mL LB medium was added to resuspend the cells. And the resuspension was incubated at 37 °C with shaking (200 rpm). Every hour from 0 to 8 h, 1 mL of the *E. coli* suspension was aliquoted, centrifuged at 4000× *g* for 10 min at 4 °C, and the supernatant was removed to obtain cell pellets. The degree of double-strand breaks in the genomic DNA was evaluated using the SFGE method.

### 4.10. Statistics and Reproducibility

Unless otherwise noted, the experiments in this study were performed with three biological replicates. A two-sided Welch’s *t*-test was used to assess significant differences between the two comparison groups, and *p* < 0.05 indicated a significant difference. Data are presented as the mean ± standard deviation.

## Data Availability

The original data presented in the study are openly available in the DNA Data Bank of Japan (DDBJ) Genomic Expression Archive (GEA) with the Experiment Accession ID E-GEAD-660.

## Supplementary Materials

The following supporting information can be downloaded at https://www.mdpi.com/article/10.3390/ijms26157275/s1.

## Abbreviations

The following abbreviations are used in this manuscript:

LB medium	Luria–Bertani medium
PBS	phosphate-buffered saline
RNA-seq	RNA sequencing
DEGs	differentially expressed genes
GO	gene ontology
KEGG	Kyoto Encyclopedia of Genes and Genomes
qRT-PCR	quantitative real-time reverse transcription polymerase chain reaction
SFGE	static field gel electrophoresis
FPKMs	fragments per kilobase of transcript per million mapped fragments
DAVID	Database for Annotation, Visualization and Integrated Discovery
CFU	Colony-forming unit
