# Supplementary material for: Acquired Radioresistance Through Adaptive Evolution with Gamma Radiation as Selection Pressure: Increased Expression and Induction of Anti-Stress Genes
Source: Int J Mol Sci. 2025 Jul 28;26(15):7275. doi: 10.3390/ijms26157275 (PMC12346926; doi:10.3390/ijms26157275)
Supplement: Supplementary file 1 [file ijms-26-07275-s001.zip › ijms-3550254-supplementary.pdf]

Supplementary Materials

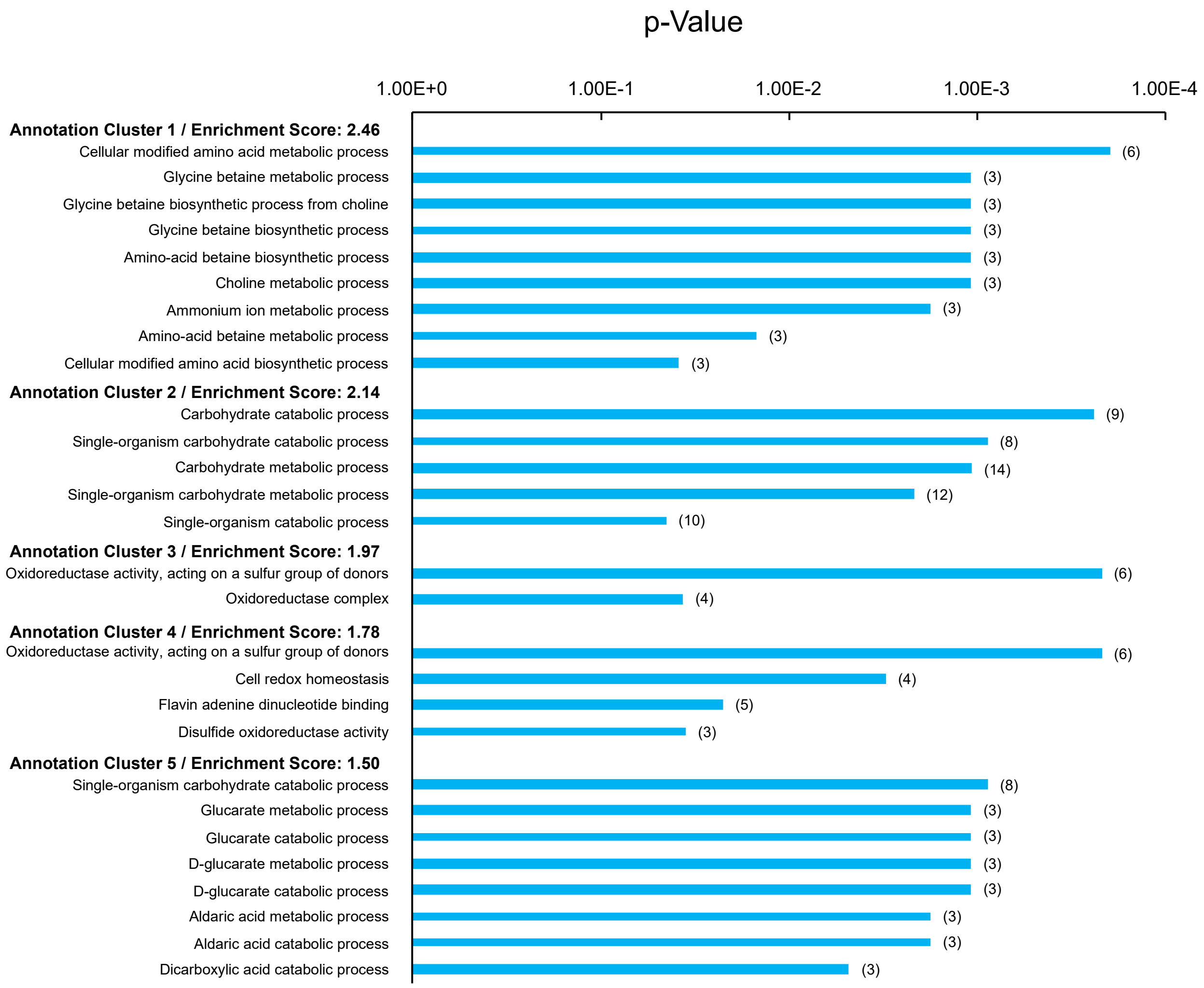

Figure S1. GO analysis of genes significantly downregulated in non-gamma-irradiated evolved *Escherichia coli* compared with those in non-gamma-irradiated wild-type *E. coli*. Vertical axis shows Annotation Cluster number, enrichment score for each Annotation Cluster, and GO terms present in each Annotation Cluster. Horizontal axis shows p-value for each GO term. Gene counts are shown in parentheses to right of bars. GO, Gene Ontology.

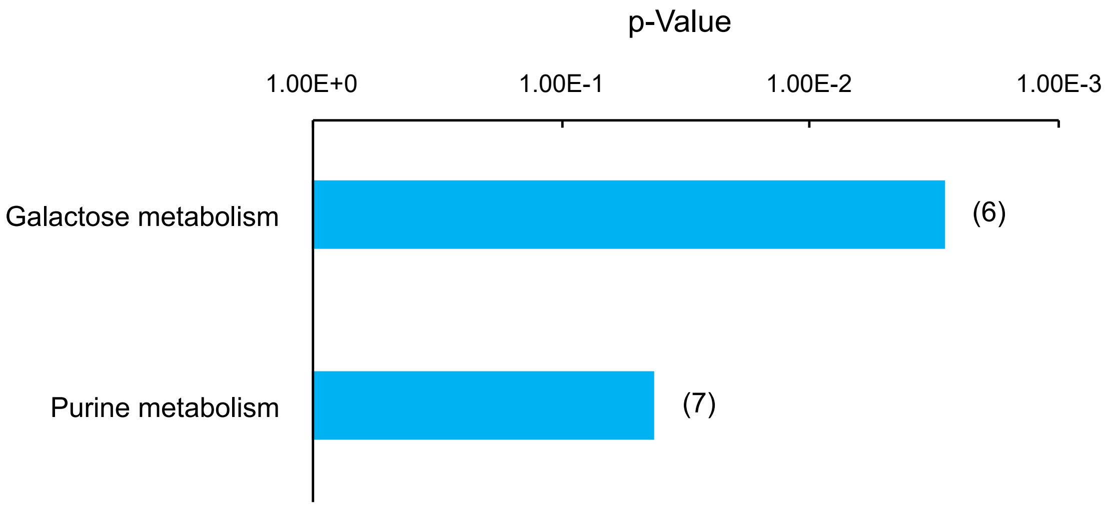

Figure S2. KEGG pathway analysis of genes significantly upregulated in non-gamma-irradiated evolved *E. coli* compared with those in non-gamma-irradiated wild-type *E. coli*. Vertical axis shows KEGG pathway terms. Horizontal axis shows p-value for each KEGG pathway term. Gene counts are shown in parentheses to right of bars. KEGG, Kyoto Encyclopedia of genes and Genomes.

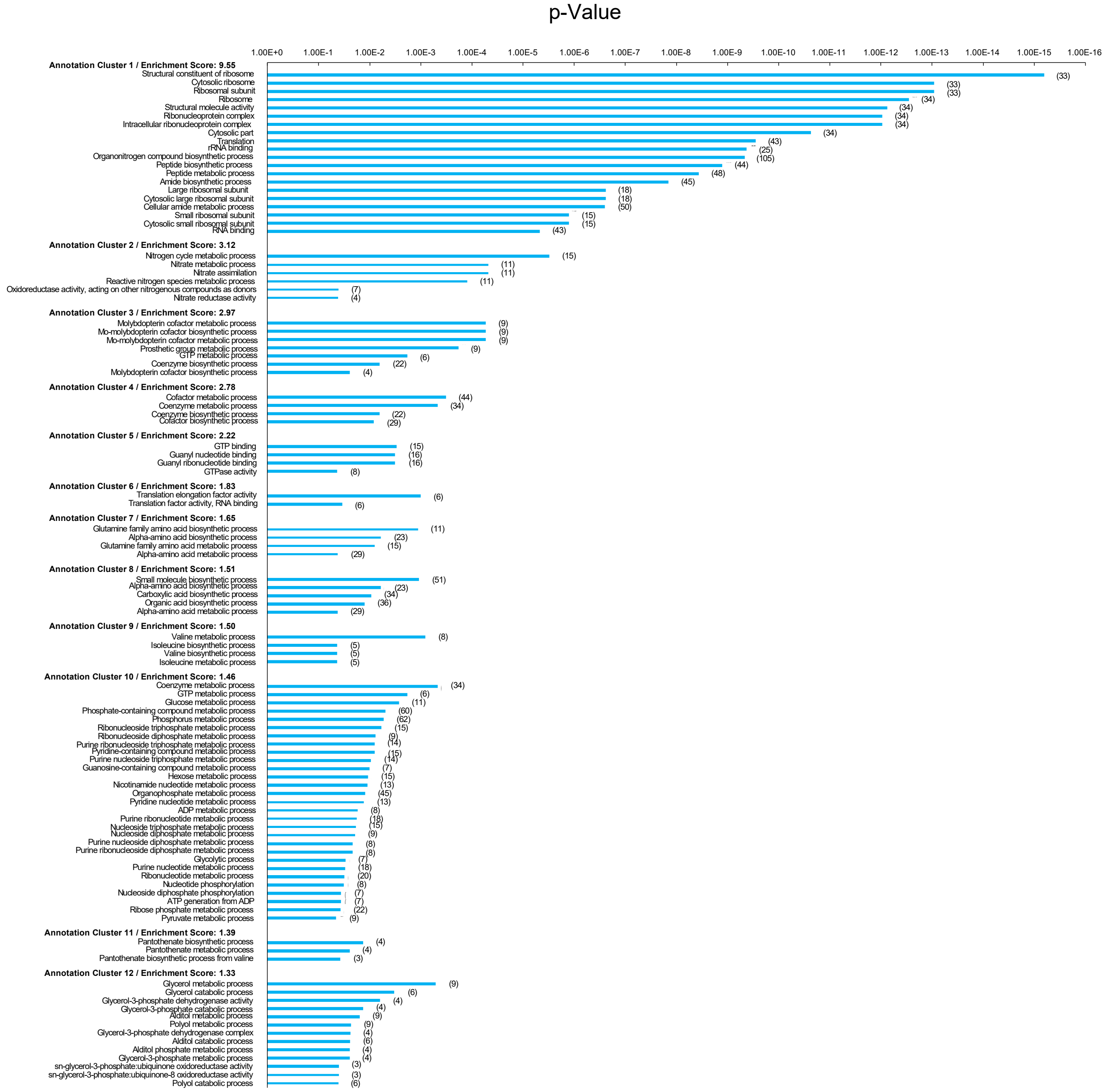

Figure S3. GO analysis of genes significantly upregulated in gamma-irradiated wild-type *E. coli* compared with those in non-gamma-irradiated wild-type *E. coli*. Vertical axis shows Annotation Cluster number, enrichment score for each Annotation Cluster, and GO terms present in each Annotation Cluster. Horizontal axis shows p-value for each GO term. Gene counts are shown in parentheses to right of bars.

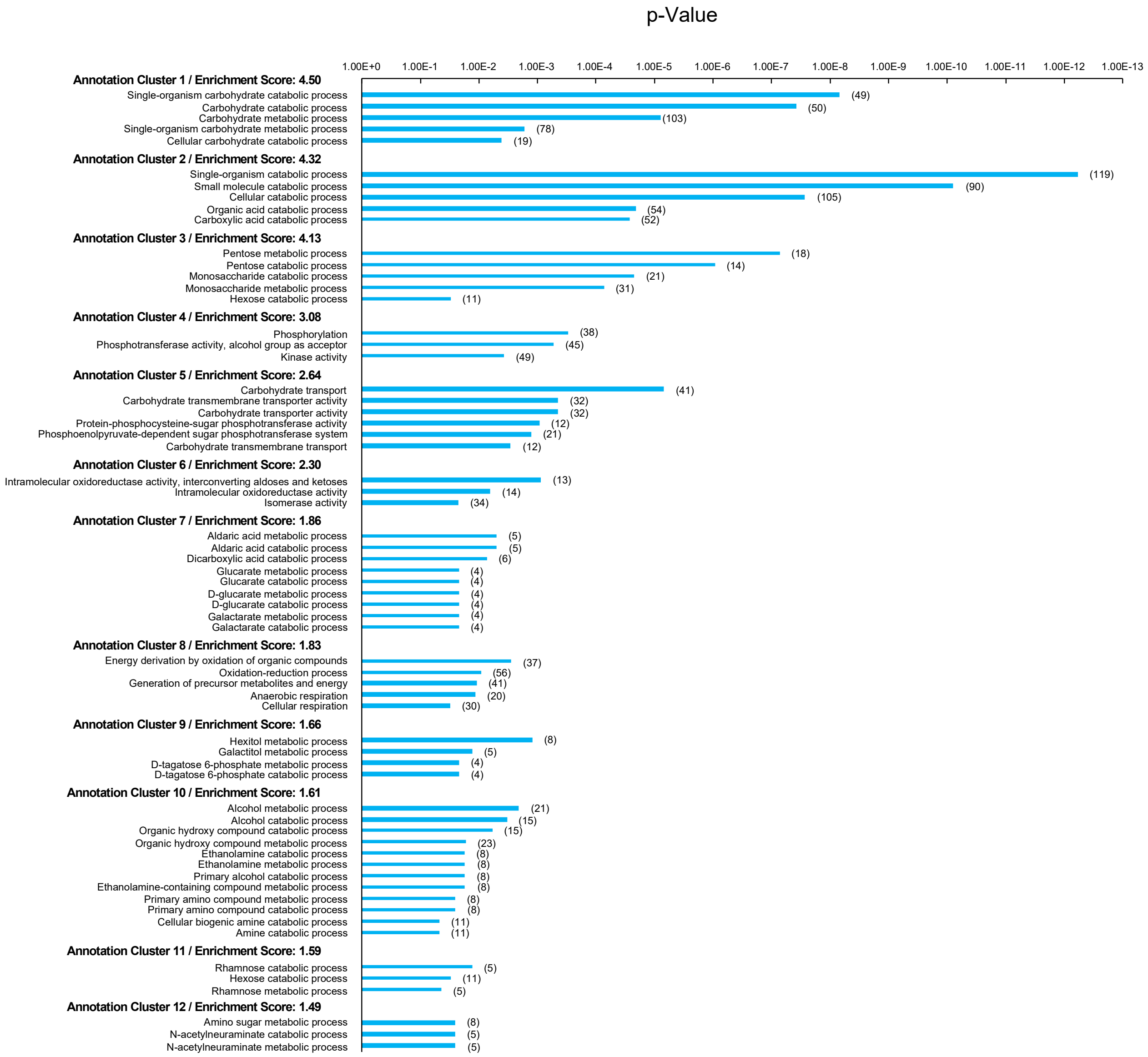

Figure S4. GO analysis of genes significantly downregulated in gamma-irradiated wild-type *E. coli* compared with those in non-gamma-irradiated wild-type *E. coli*. Vertical axis shows Annotation Cluster number, enrichment score for each Annotation Cluster, and GO terms present in each Annotation Cluster. Horizontal axis shows p-value for each GO term. Gene counts are shown in parentheses to right of bars.

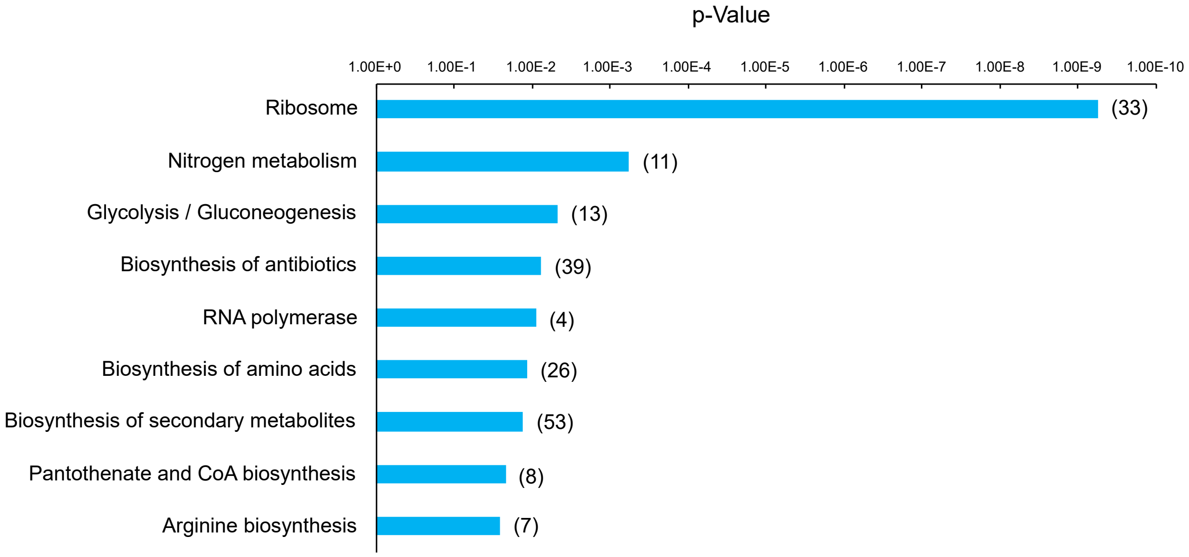

Figure S5. KEGG pathway analysis of genes significantly upregulated in gamma-irradiated wild-type *E. coli* compared with those in non-gamma-irradiated wild-type *E. coli*. Vertical axis shows KEGG pathway terms. Horizontal axis shows p-value for each KEGG pathway term. Gene counts are shown in parentheses to right of bars.

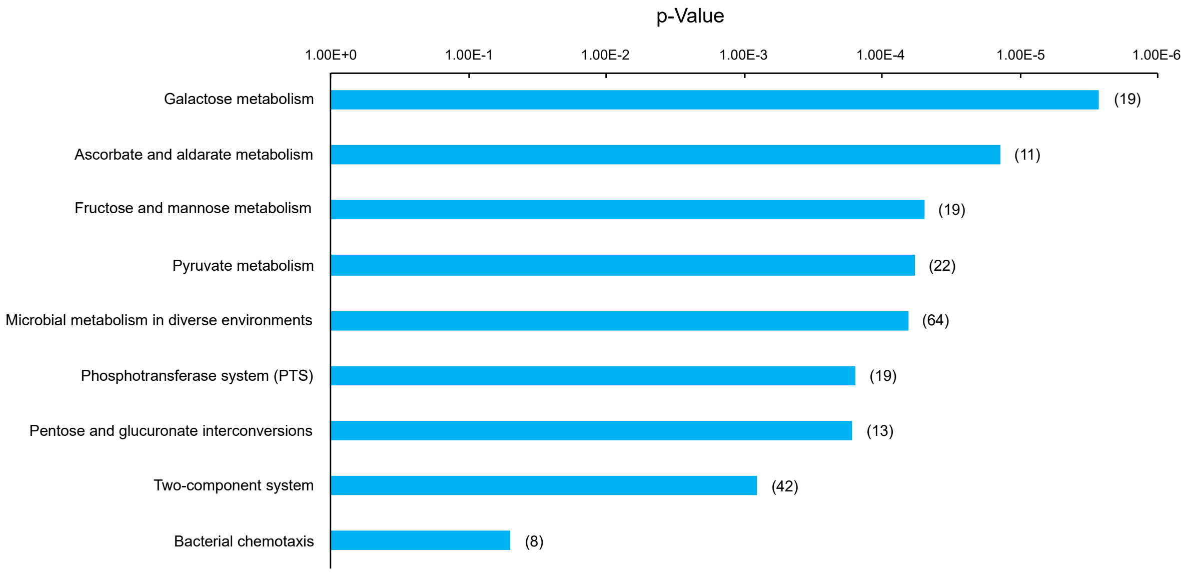

Figure S6. KEGG pathway analysis of genes significantly downregulated in gamma-irradiated wild-type *E. coli* compared with those in non-gamma-irradiated wild-type *E. coli*. Vertical axis shows KEGG pathway terms. Horizontal axis shows p-value for each KEGG pathway term. Gene counts are shown in parentheses to right of bars.

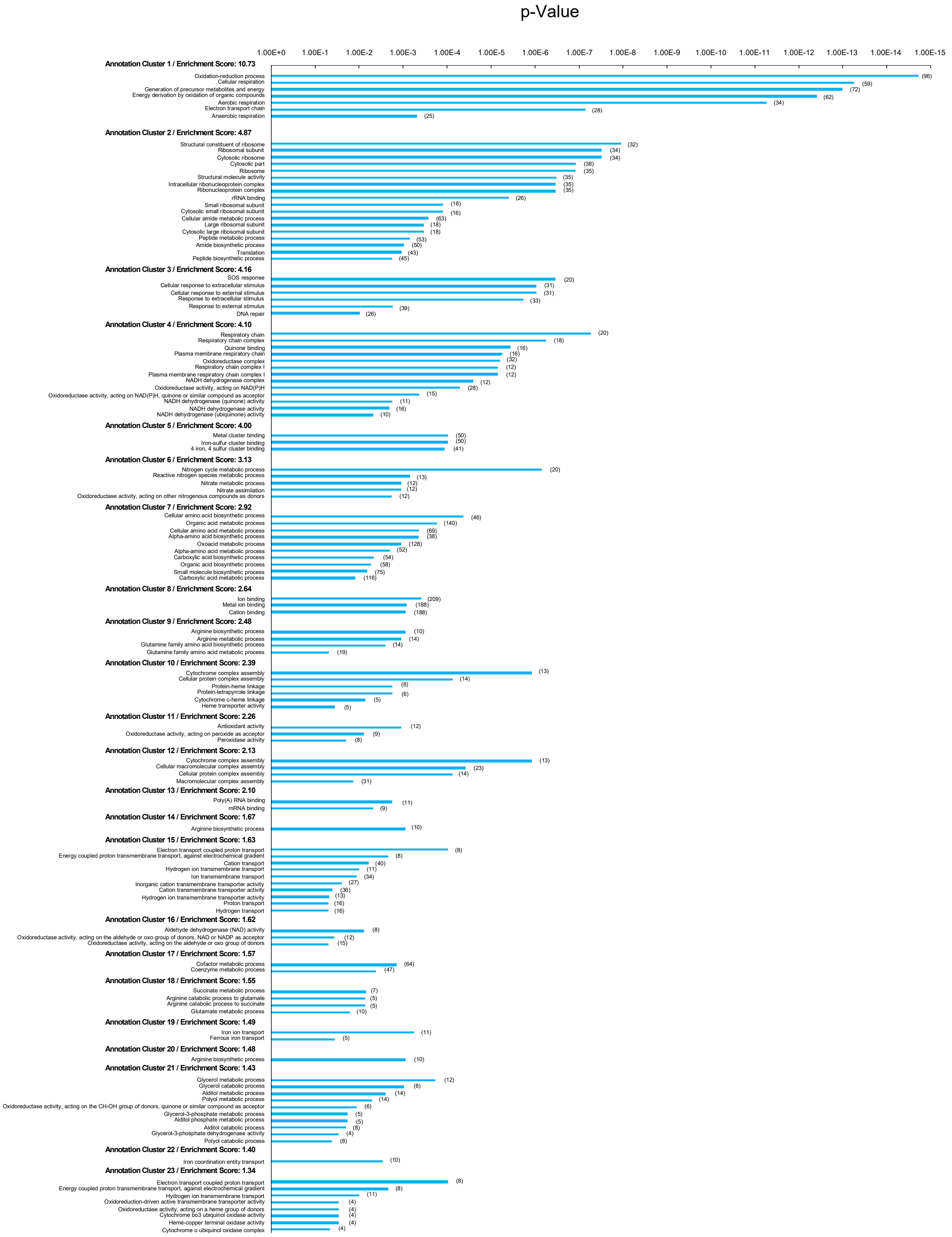

Figure S7. GO analysis of genes significantly upregulated in gamma-irradiated evolved *E. coli* compared with those in non-gamma-irradiated evolved *E. coli*. Vertical axis shows Annotation Cluster number, enrichment score for each Annotation Cluster, and GO terms present in each Annotation Cluster. Horizontal axis shows p-value for each GO term. Gene counts are shown in parentheses to right of bars.

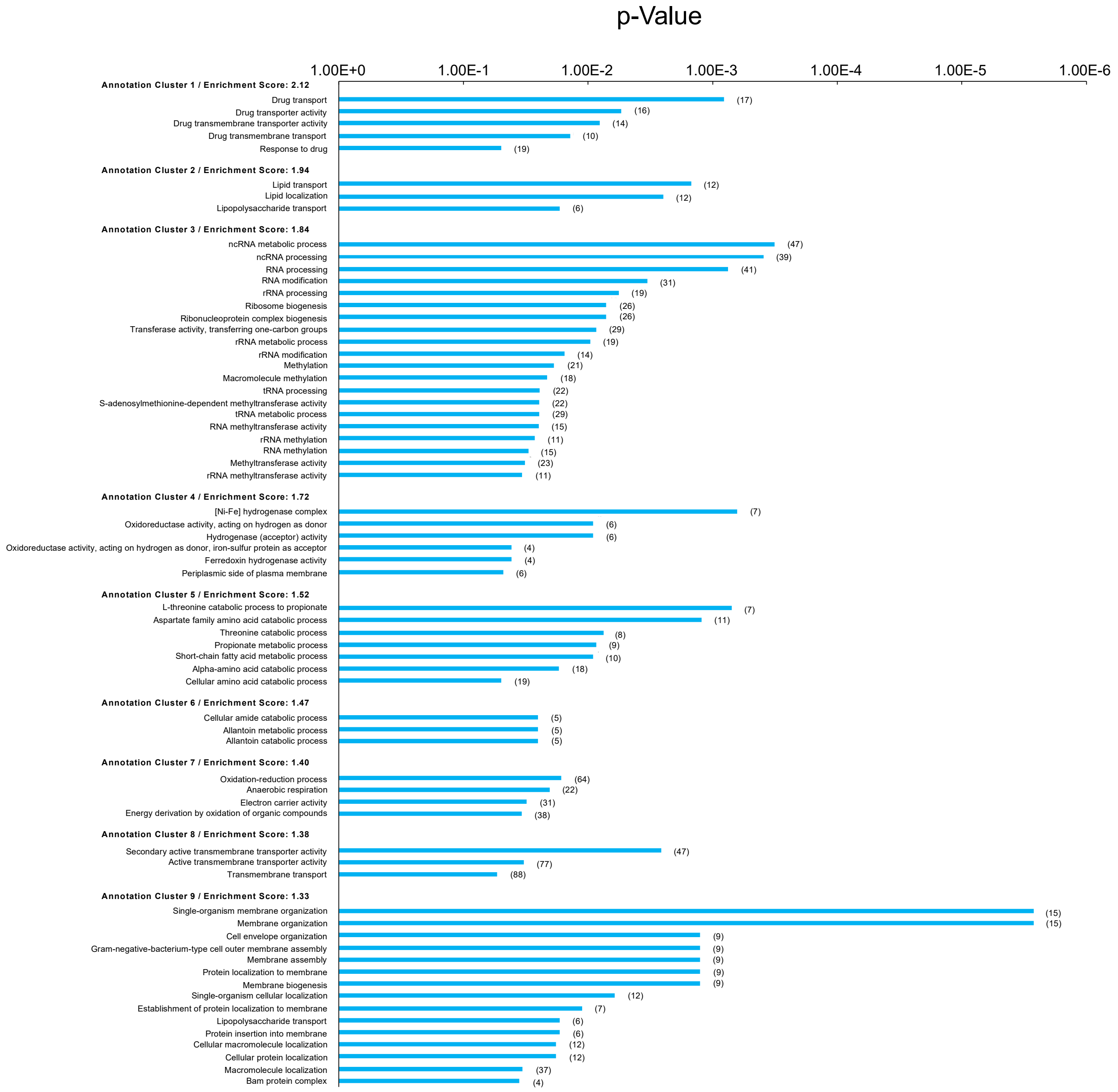

Figure S8. GO analysis of genes significantly downregulated in gamma-irradiated evolved *E. coli* compared with those in non-gamma-irradiated evolved *E. coli*. Vertical axis shows Annotation Cluster number, enrichment score for each Annotation Cluster, and GO terms present in each Annotation Cluster. Horizontal axis shows p-value for each GO term. Gene counts are shown in parentheses to right of bars.

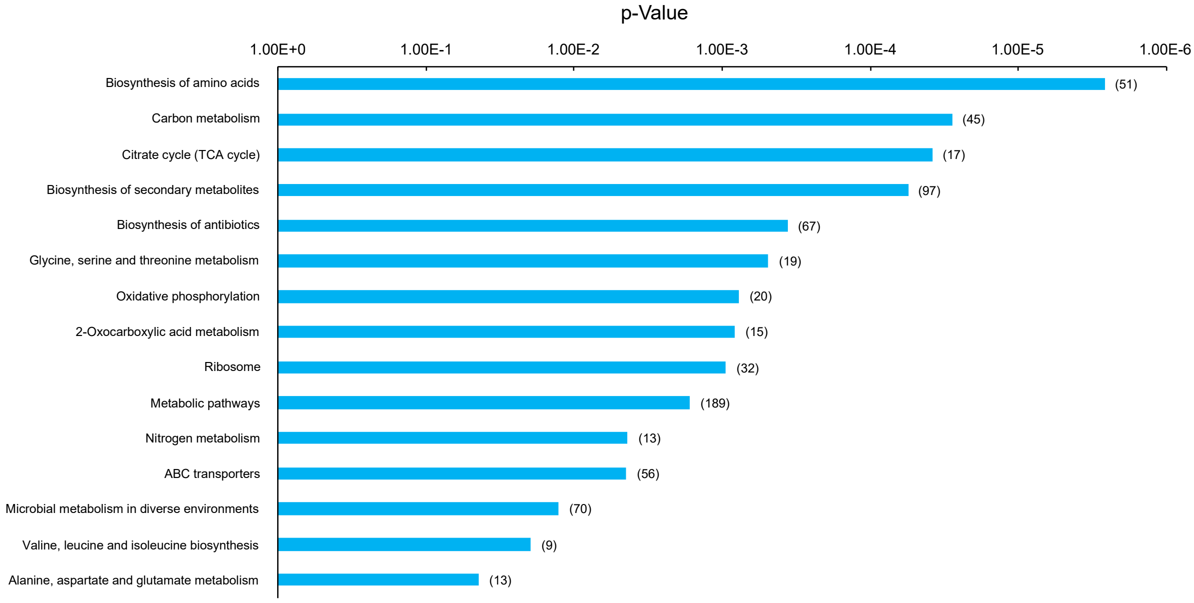

Figure S9. KEGG pathway analysis of genes significantly upregulated in gamma-irradiated evolved *E. coli* compared with those in non-gamma-irradiated evolved *E. coli*. Vertical axis shows KEGG pathway terms. Horizontal axis shows p-value for each KEGG pathway term. Gene counts are shown in parentheses to right of bars.

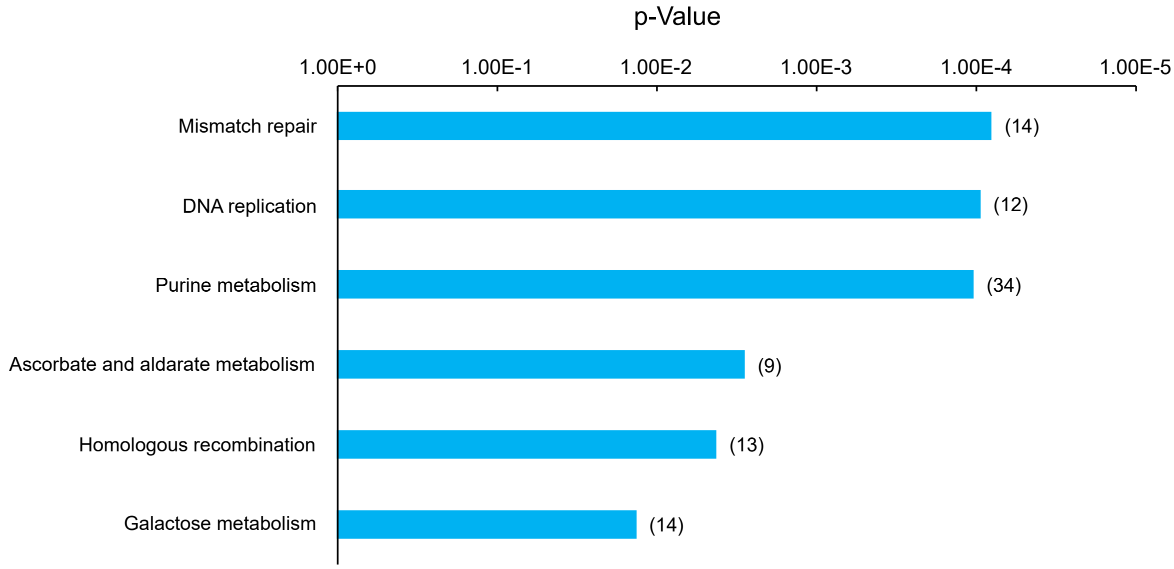

Figure S10. KEGG pathway analysis of genes significantly downregulated in gamma-irradiated evolved *E. coli* compared with those in non-gamma-irradiated evolved *E. coli*. Vertical axis shows KEGG pathway terms. Horizontal axis shows p-value for each KEGG pathway term. Gene counts are shown in parentheses to right of bars.

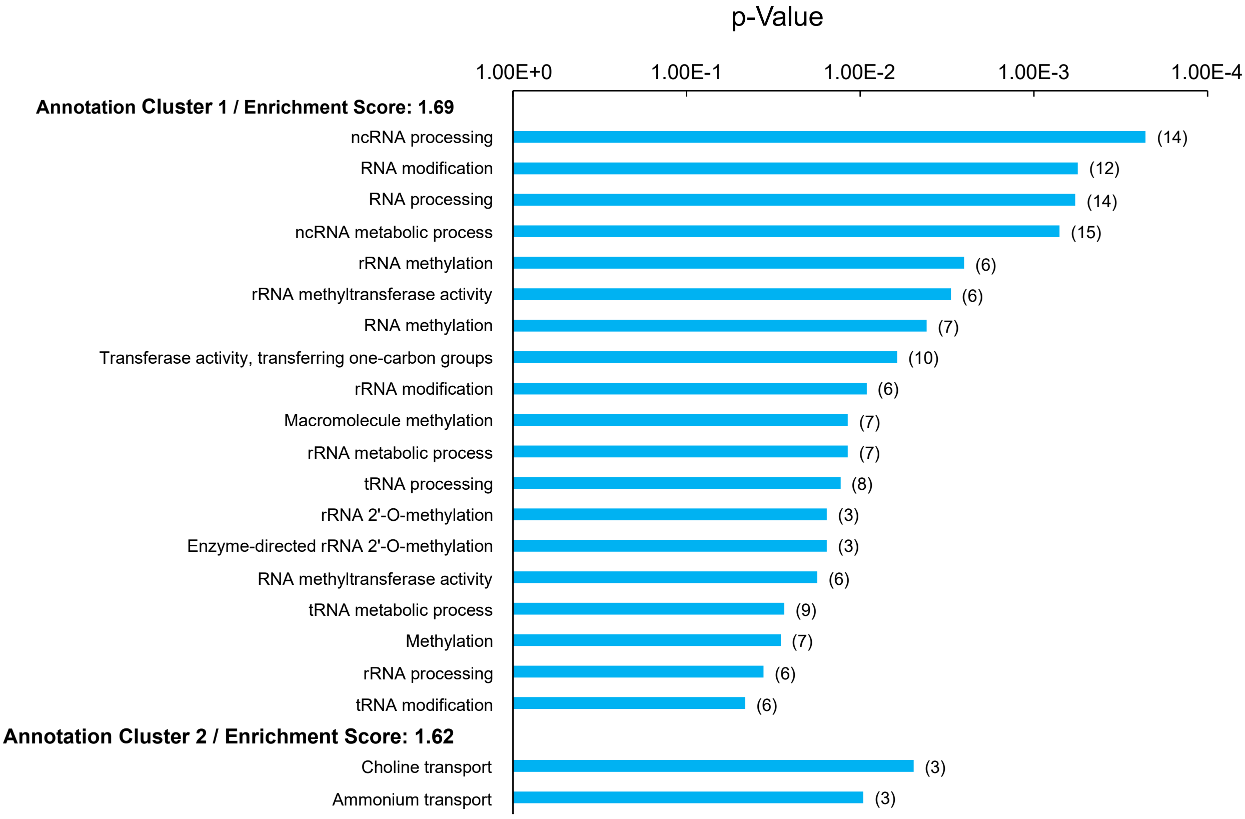

Figure S11. GO analysis of genes significantly downregulated in gamma-irradiated evolved *E. coli* compared with those in gamma-irradiated wild-type *E. coli*. Vertical axis shows Annotation Cluster number, enrichment score for each Annotation Cluster, and GO terms present in each Annotation Cluster. Horizontal axis shows p-value for each GO term. Gene counts are shown in parentheses to right of bars.

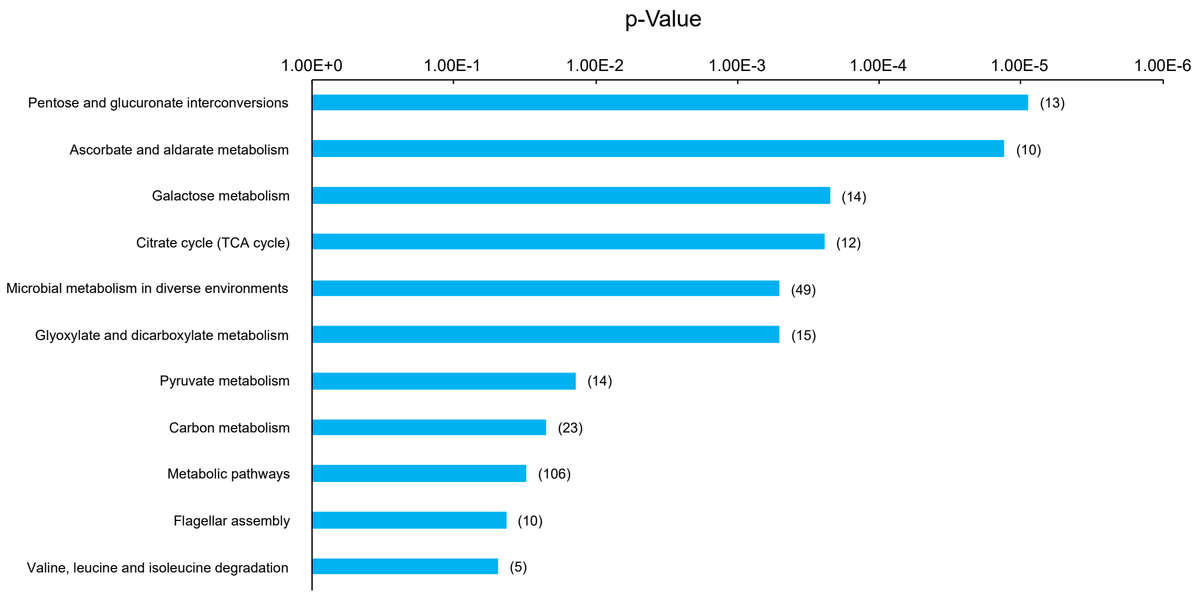

Figure S12. KEGG pathway analysis of genes significantly upregulated in gamma-irradiated evolved *E. coli* compared with those in gamma-irradiated wild-type *E. coli*. Vertical axis shows KEGG pathway terms. Horizontal axis shows p-value for each KEGG pathway term. Gene counts are shown in parentheses to right of bars.

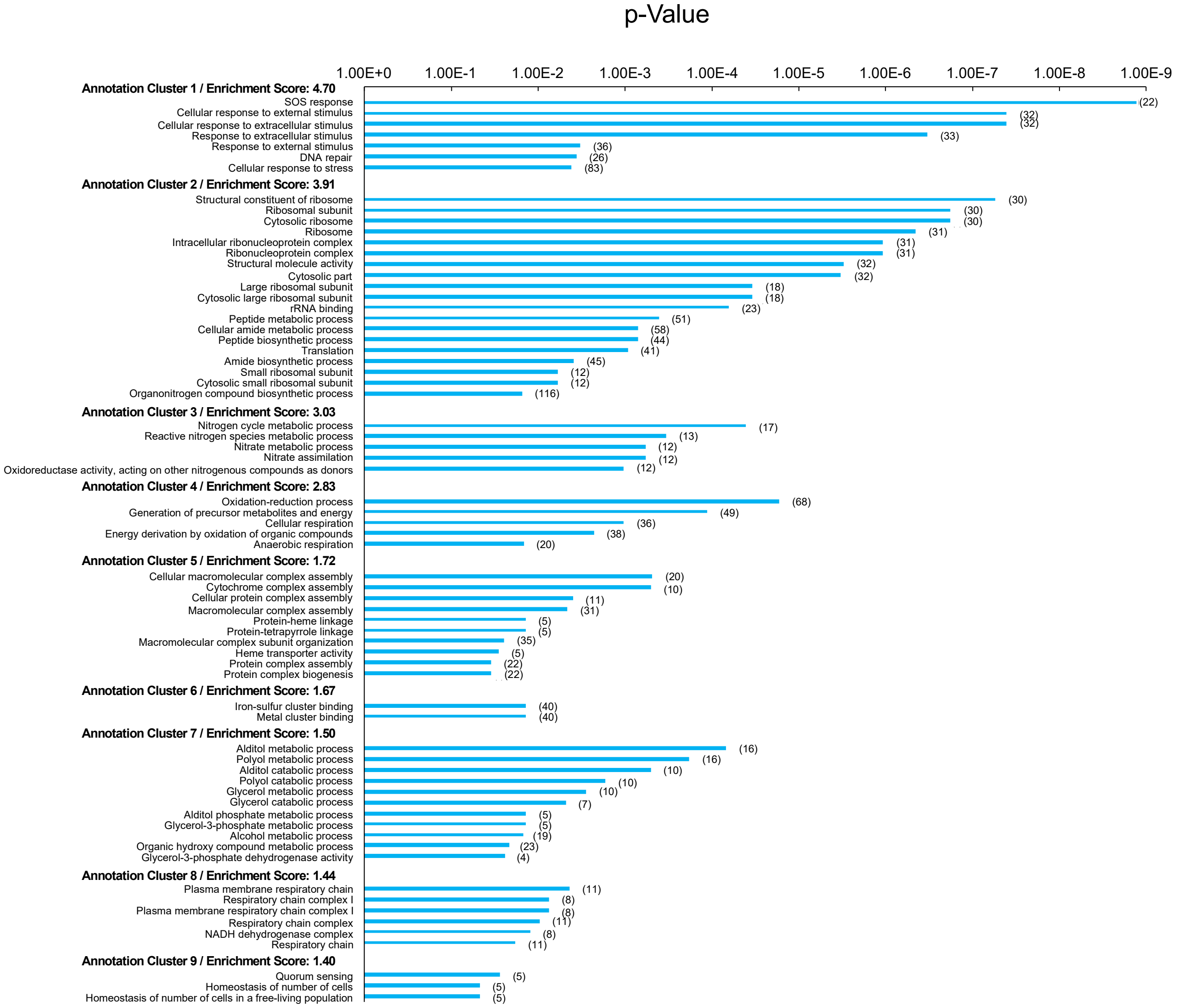

Figure S13. GO analysis of genes significantly upregulated in gamma-irradiated evolved *E. coli* compared with those in non-gamma-irradiated wild-type *E. coli*. Vertical axis shows Annotation Cluster number, enrichment score for each Annotation Cluster, and GO terms present in each Annotation Cluster. Horizontal axis shows p-value for each GO term. Gene counts are shown in parentheses to right of bars.

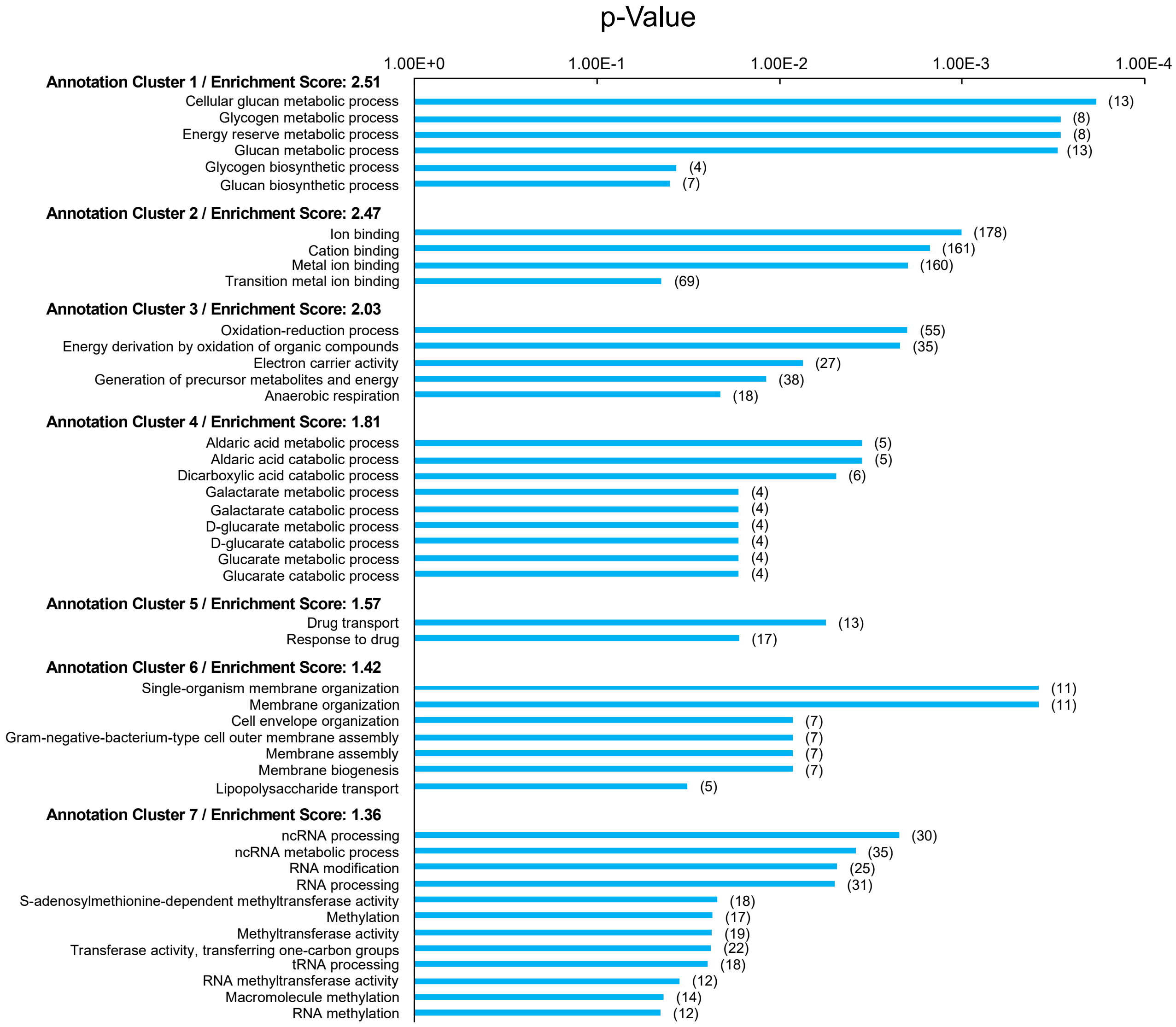

Figure S14. GO analysis of genes significantly downregulated in gamma-irradiated evolved *E. coli* compared with those in non-gamma-irradiated wild-type *E. coli*. Vertical axis shows Annotation Cluster number, enrichment score for each Annotation Cluster, and GO terms present in each Annotation Cluster. Horizontal axis shows p-value for each GO term. Gene counts are shown in parentheses to right of bars.

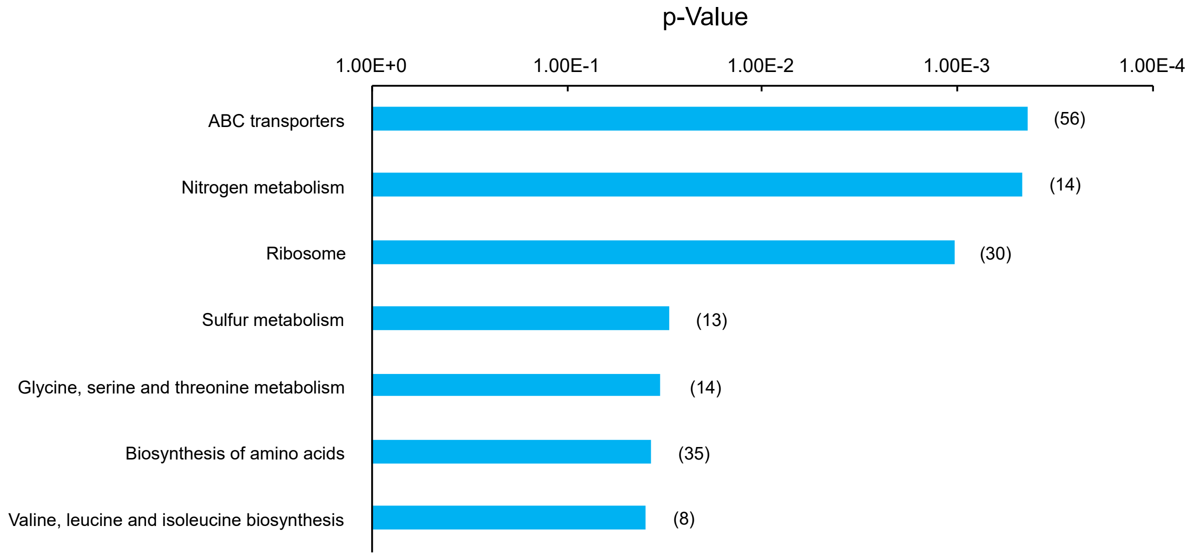

Figure S15. KEGG pathway analysis of genes significantly upregulated in gamma-irradiated evolved *E. coli* compared with those in non-gamma-irradiated wild-type *E. coli*. Vertical axis shows KEGG pathway terms. Horizontal axis shows p-value for each KEGG pathway term. Gene counts are shown in parentheses to right of bars.

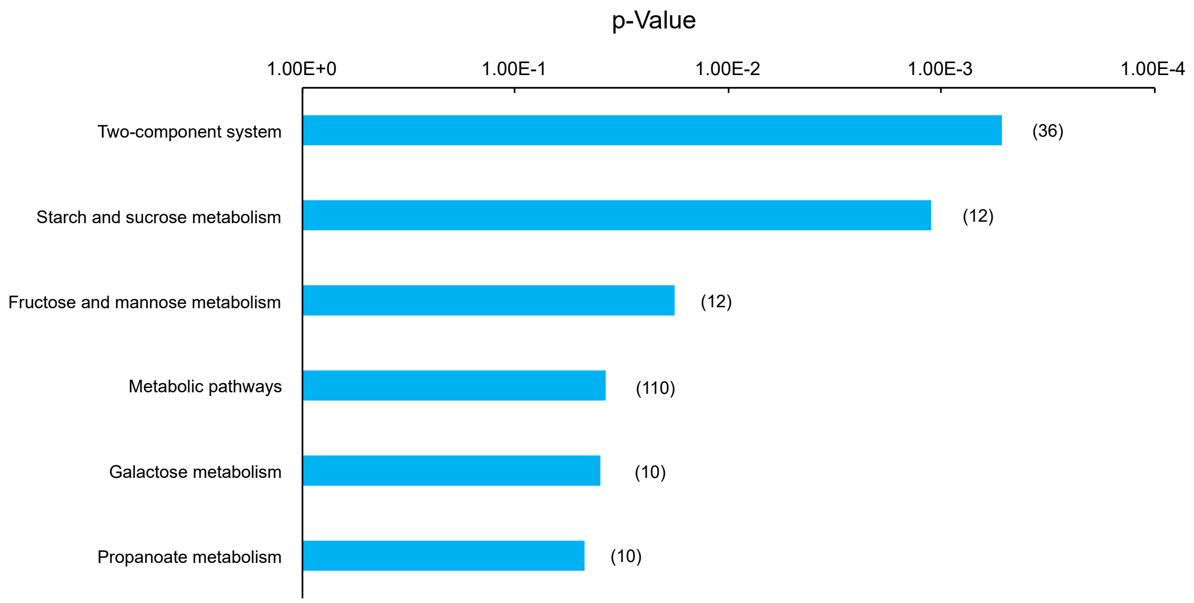

Figure S16. KEGG pathway analysis of genes significantly downregulated in gamma-irradiated evolved *E. coli* compared with those in non-gamma-irradiated wild-type *E. coli*. Vertical axis shows KEGG pathway terms. Horizontal axis shows p-value for each KEGG pathway term. Gene counts are shown in parentheses to right of bars.

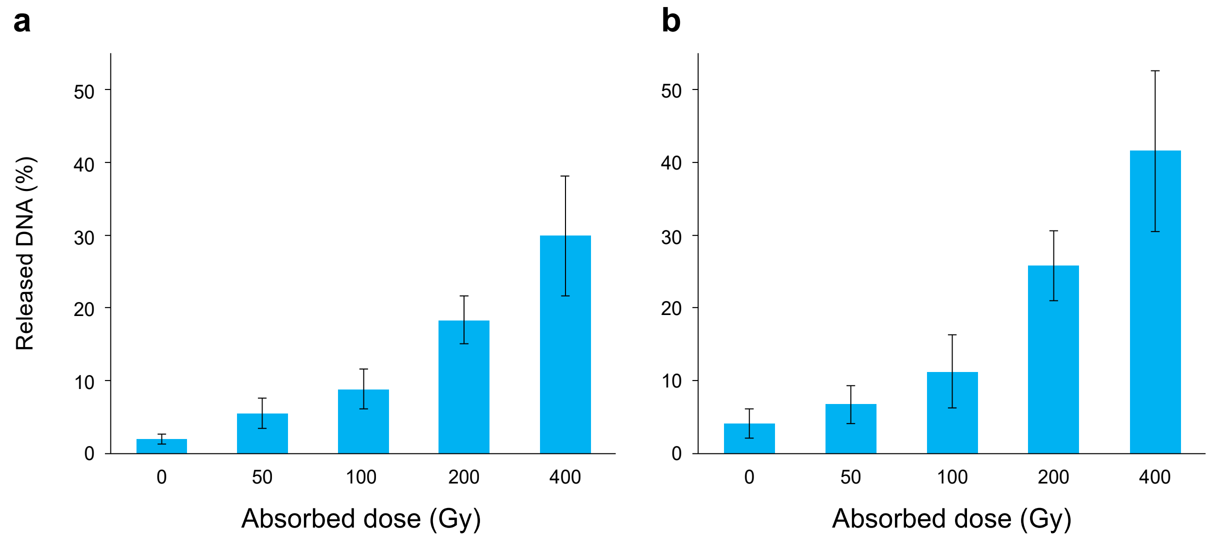

Figure S17. Acute genomic DNA damage after gamma irradiation**. a)** DNA damage in wild-type *E. coli*; **b)** DNA damage in evolved *E. coli*. Degree of double-strand breaks in genomic DNA of cells irradiated with 0, 50, 100, 200, and 400 Gy of gamma radiation was evaluated via Static Field Gel Electrophoresis method. Horizontal axis shows absorbed dose of gamma radiation, and vertical axis shows proportion of DNA released from well relative to total DNA.

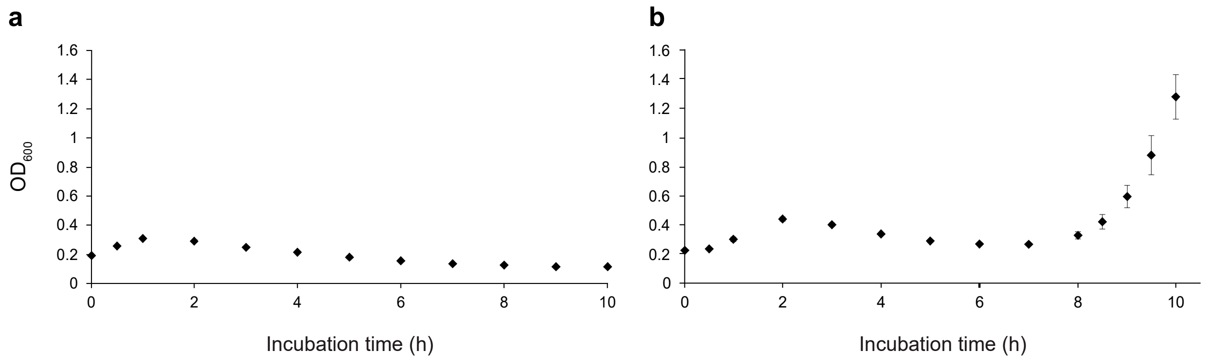

Figure S18. Growth time course after gamma irradiation. **a)** Growth of wild-type *E. coli*; **b)** Growth of evolved *E. coli*. Cells irradiated with 2.5 kGy of gamma radiation were shake-incubated in LB medium at 37 °C. Turbidity of incubated suspension was measured at each incubation time. Horizontal axis shows incubation time, vertical axis shows turbidity (optical density) of cell suspension measured at 600 nm.

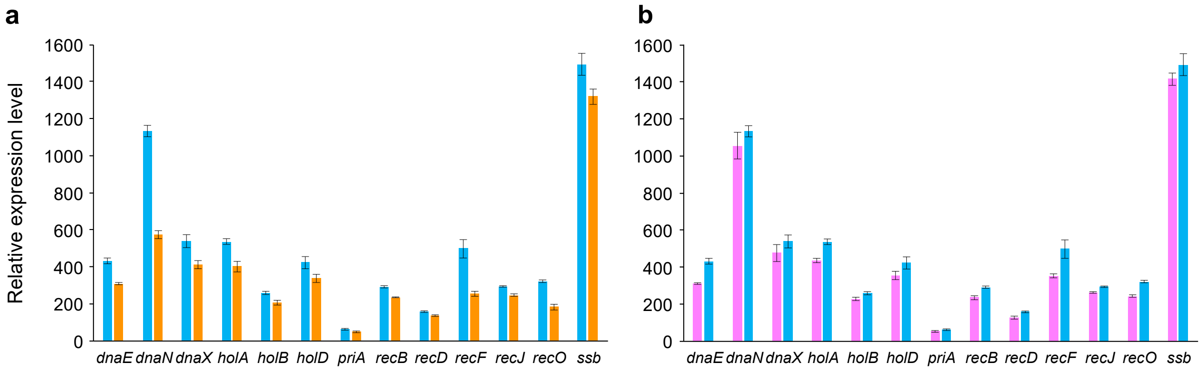

Figure S19. Relative expression levels of homologous recombination-related genes. Relative expression levels of homologous recombination-related genes enriched in KEGG pathway analysis of genes whose expression was downregulated in gamma-irradiated evolved *E. coli* compared with non-gamma-irradiated evolved *E. coli* were compared. **a)** Non-gamma-irradiated evolved versus gamma-irradiated evolved *E. coli*. Blue bars indicate results for non-gamma-irradiated evolved *E. co*li, and or*ange* bars indicate those for gamma-irradiated evolved *E. coli*; **b)** Non-gamma-irradiated wild-type versus non-gamma-irradiated evolved *E. coli*. Pink bars indicate results for non-gamma-irradiated wild-type *E. coli,* blue bars indicate those for non-gamma-irradiated evolved *E. coli*. Horizontal axis shows genes, vertical axis shows relative expression level of each gene.

Table S1. Genes involved in GO term in Figure 4.

Annotation Cluster 1/Enrichment Score: 2.43

Term	Genes
SOS response	*sulA, ydjM, recQ, umuC, umuD, yafP, dinB, rimK*
Cellular response to extracellular stimulus	*sulA, ydjM, recQ, umuC, umuD, yhjX, yafP, dinB, rimK*
Cellular response to external stimulus	*sulA, ydjM, recQ, umuC, umuD, yhjX, yafP, dinB, rimK*
Response to extracellular stimulus	*sulA, ydjM, recQ, umuC, umuD, yhjX, yafP, dinB, rimK*
DNA repair	*ycjD, recO, sulA, recQ, umuC, umuD, yafP, dinB*
DNA metabolic process	*ycjD, nrdD, sulA, recO, dnaE, recQ, umuC, umuD, sbmC, insQ, rmuC, holA, endA, yafP, dinB*
Response to external stimulus	*sulA, ydjM, recQ, umuC, umuD, yhjX, yafP, dinB, rimK*

Annotation Cluster 2/Enrichment Score: 1.72

Term	Genes
DNA-directed DNA polymerase activity	*dnaE, umuD, dinB, holA*
DNA polymerase activity	*dnaE, umuD, dinB, holA*
DNA biosynthetic process	*dnaE, umuD, dinB*

Table S2. Genes involved in GO terms of Annotation Cluster 1 in Figure 5.

Annotation Cluster 1/Enrichment Score: 7.25

Term	Genes
SOS response	*recN, ruvA, dinG, cho, uvrD, sulA, lexA, ydjM, umuC, uvrA, yafN, ruvB, dinD, recX, yebG, rimK, uvrB, dinI, recA, yafP, polB, dinB, yafO*
Cellular response to external stimulus	*recN, ahpC, cho, yjiY, ydjM, umuC, uvrA, ruvB, dinD, rimK, uvrB, dinI, dinB, ruvA, dinG, uvrD, sulA, lexA, ykgM, cstA, rhsD, appA, yafN, yhjX, recX, zinT, yebG, recA, yafP, polB, yafO*
Cellular response to extracellular stimulus	*recN, ahpC, cho, yjiY, ydjM, umuC, uvrA, ruvB, dinD, rimK, uvrB, dinI, dinB, ruvA, dinG, uvrD, sulA, lexA, ykgM, cstA, rhsD, appA, yafN, yhjX, recX, zinT, yebG, recA, yafP, polB, yafO*
Response to extracellular stimulus	*recN, ahpC, cho, yjiY, ydjM, umuC, uvrA, ruvB, csiD, dinD, rimK, uvrB, dinI, dinB, ruvA, dinG, uvrD, sulA, lexA, ykgM, cstA, rhsD, appA, yafN, yhjX, recX, zinT, yebG, recA, yafP, polB, yafO*
Response to external stimulus	*recN, ahpC, cho, tar, yjiY, ydjM, umuC, uvrA, ruvB, acs, csiD, dinD, rimK, uvrB, dinI, mglB, dinB, ruvA, dinG, trg, uvrD, sulA, lexA, ykgM, cstA, rhsD, appA, yafN, yhjX, mazF, recX, rbsB, zinT, yebG, dppA, recA, yafP, polB, yafO*
Cellular response to DNA damage stimulus	*glxR, asnA, umuC, thiH, idi, ydiZ, dsdX, pfkB, recB, recD, uvrB, dinI, yiaO, uxuA, yjhC, dinB, paoC, eutN, sbmC, yafN, nanK, nagE, ygjK, yhcH, htpG, yjiX, yafP, yafO, recN, yaaY, cho, yjiY, ydjM, uvrA, ruvB, dinD, flgI, dinG, ycjD, ruvA, uvrD, sulA, mglC, glpK, lexA, hisA, otsA, glgA, recX, yebG, mglA, recA, polB, dsdA*
DNA repair	*recN, ycjD, ruvA, dinG, cho, uvrD, sulA, lexA, umuC, uvrA, yafN, ruvB, recX, yebG, recB, uvrB, dinI, recD, recA, yafP, polB, dinB, yafO*
Cellular response to stress	*glxR, ahpC, asnA, umuC, thiH, idi, ydiZ, dsdX, pfkB, recB, recD, uvrB, dinI, yiaO, uxuA, yjhC, dinB, paoC, cstA, ykgM, eutN, kdpD, sbmC, yafN, nanK, nagE, ygjK, yhcH, htpG, yjiX, yafP, yafO, recN, yaaY, cho, yjiY, rmf, ydjM, uvrA, ruvB, dinD, treF, rimK, flgI, dinG, ycjD, ruvA, uvrD, sulA, mglC, glpK, lexA, hisA, otsA, rhsD, glgA, appA, recX, zinT, yebG, mglA, osmF, recA, polB, dsdA*

Table S3. Assay IDs, primer sequences, probe sequences, fluorophore, and quencher for Custom TaqMan Gene Expression Assays.

Gene	Assay ID	Forward primer sequence	Reverse primer sequence	Probe sequence	Fluorophore/ Quencher
yhjX	AP329G3	CAGGAAGTGAAAACCAGCAATGG	GCGGTTTACGCATCGACTCT	CAGCGTGTAATCTTTC	FAM/NFQ*
sulA	APU69MW	CTCTGGTTAACACCGCAACAAAA	GGAGAGCTGGCTAATCTGCATT	CCAGATGCCTGAACCC	FAM/NFQ*
umuC	AP4722Z	TTTATCCGTAAACATTTTAATGTCGTGCTC	GTTCGCCGCGCAGTTC	ACGCACCGTTCTTTC	FAM/NFQ*
ydjM	AP7DWMX	GCTGCTGGCAACCTTTTACC	ACCCAGCACCATTCCTTGTAG	CAGCCGGAATGAACC	FAM/NFQ*
dinB	AP9HP7V	CAACGGCCAGTTTGTGATTACG	TTGCCAGCGGTAAGGTTTGTA	AAAATGCCGGAACTTC	FAM/NFQ*
recA	APT2E2Y	CTTGCGGCACGTATGATGAG	CGTGTTGGACTGCTTCAGGTT	ACCCGCCAGCTTAC	FAM/NFQ*
gyrA	APRWKG2	TGCGTGATGGTCTGTACTACCT	GCAAACGCAGATCCAGAATCG	CCGAACAGCAAGCTCA	FAM/NFQ*

* FAM: 6-carboxyfluorescein, NFQ: nonfluorescent quencher
